# Differences in Gene Expression Profiles and Phenotypes of Differentiated SH-SY5Y Neurons Stably Overexpressing Mitochondrial Ferritin

**DOI:** 10.3389/fnmol.2018.00470

**Published:** 2019-01-08

**Authors:** Anarmaa Mendsaikhan, Shigeko Takeuchi, Douglas G. Walker, Ikuo Tooyama

**Affiliations:** Molecular Neuroscience Research Center, Shiga University of Medical Science, Otsu, Japan

**Keywords:** neuroprotection, free radical, mitochondria, iron, neurodegeneration, cell culture

## Abstract

Mitochondrial ferritin (FtMt) is an iron-transport protein with ferroxidase properties localized to mitochondria. Levels are generally low in all tissues, while increasing the expression of FtMt in neuronal-like cells has been shown to be protective. To determine whether FtMt has potential as a therapeutic approach, there remains the question of how much FtMt is protective. To address this issue, we transfected SH-SY5Y neuroblastoma cells with a FtMt expression plasmid and isolated cell lines with stable expression of FtMt at high, medium and low levels. Using these cell lines, we examined effects of FtMt on neuronal phenotype, neuroprotective activity and gene expression profiles. The phenotypic properties of high, medium and low FtMt expressors were compared with native untransfected SH-SY5Y cells after differentiation with retinoic acid to a neuronal phenotype. Overexpression of FtMt, even in low expressing cells, showed significant protection from oxidative stress induced by hydrogen peroxide or cobalt chloride. Higher levels of FtMt expression did not appear to offer greater protection, and did not have toxic consequences to cells, even though there were significantly more aggregated mitochondria in the highest expressing clone. The phenotypes differed between cell clones when assessed by cell growth, neurite outgrowth, and expression of neuronal proteins including those associated with neurodegenerative diseases. Microarray analysis of high, medium and negative FtMt-expressing cells identified different patterns of expression of certain genes associated with oxidative stress and neuronal development, amongst others. Validation of microarray analyses was carried out by real time polymerase chain reaction. The results showed significant differences in expression of thioredoxin-interacting protein (TXNIP) and microsomal glutathione transfer-1 (MGST-1), which can have critical roles in the regulation of oxidative stress. Differences in expression of calcitonin-related polypeptide alpha (CALCA), growth differentiation factor-15 (GDF-15) and secretogranin II (SCG2) were also observed. Our findings indicate that even low levels of increased FtMt expression can be protective possibly by alterations of some oxidative stress-related and growth factor genes, while high levels of expression did not appear to offer greater protection from oxidative stress or induce significant toxicity in cells. These experiments provide supporting data that increasing FtMt might be a feasible strategy for therapeutics in certain neurodegenerative and neurological diseases.

## Introduction

Age-associated neurodegenerative diseases, particularly Alzheimer's disease (AD) and Parkinson's disease (PD), have proven resistant to effective therapies as the pathological processes are complex and still incompletely understood. There have been multiple approaches aimed at slowing down the degenerative processes, but they have primarily focused on inhibiting the formation or promoting the removal of toxic forms of amyloid beta (Aβ) peptide or tau (for AD) or α-synuclein (for PD) (Brundin et al., 2017; Jan et al., 2017). Although these diseases have significant clinical and pathological differences, one common feature is increased oxidative stress through elevated levels of damaging reactive oxygen species (ROS) (Nesi et al., 2017; Lang and Espay, 2018). Effective regulation of intracellular iron plays a critical role in controlling generation of ROS. Ferritin-H is the major cellular regulator of iron in most cells, but a significant role has been identified for mitochondrial ferritin (FtMt), a ferritin-H-like molecule that is targeted to mitochondria (Arosio et al., 2009).

FtMt has roles that overlap with ferritin, but its expression in normal tissue is very low and restricted to testis, brain, heart, and erythroblasts (Levi and Arosio, 2004; Santambrogio et al., 2007). FtMt expression is highest in cells of tissues with high oxygen demand, including neurons in brain (Gao and Chang, 2014). FtMt has multiple properties, in particular ferroxidase activity to reduce reactive ferrous ions to less reactive ferric ions. Within the realm of biological processes, it participates in oxidation-reduction, iron transport across membranes and cellular iron homeostasis. FtMt is synthesized as a 30 kDa polypeptide that is cleaved once the protein is translocated to the mitochondria matrix and assembles into a “ferritin-shell” of 24 of 22 kDa polypeptide chains (Levi et al., 2001; Drysdale et al., 2002). FtMt binds iron with similar properties to ferritin, but has particularly important function of regulating reactive iron species in mitochondria (Yang et al., 2013; Wang Y. Q. et al., 2016). In contrast to ferritin, FtMt mRNA lacks an iron-response element indicating a different mechanism of regulation (Drysdale et al., 2002). Recently, the FtMt gene promoter was shown to contain positive regulatory sequences for cyclic-AMP response element-binding protein (CREB), YYI and SP1 transcription factors, and C/EBPβ, GATA2 and FOXA1 sequences as negative regulators (Guaraldo et al., 2016).

With reference to the involvement of FtMt in diseases, it has been associated with Friedreich's Ataxia, restless leg syndrome and macular degeneration (Huang et al., 2009; Snyder et al., 2009; Stenirri et al., 2012; Wang X. et al., 2016). Overexpression of FtMt significantly slowed the replication of FtMt transfected SH-SY5Y neuroblastoma cells, both in culture and after *in vivo* transplantation of overexpressing cells to immune-deficient mice (Gong et al., 2017). Increased expression of FtMt has been demonstrated in neurons in regions of human brains affected by AD and PD pathology (Wang et al., 2011; Yang et al., 2017).

A number of studies using overexpression or knockdown models employing neuronal-like cells, particularly SH-SY5Y cells, demonstrated that FtMt protected against oxidative stressors and Aβ neurotoxicity (Shi et al., 2015; Gao et al., 2017; Li X. et al., 2017; Wang et al., 2017), (Wu et al., 2013; Wang Y. Q. et al., 2016; Gao et al., 2017; Guan et al., 2017). The potential therapeutic benefits of FtMt have also been suggested from different animal models for AD or PD. Using a line of mice with deletion of FtMt gene, it was shown that intracerebroventricular administration of the toxic Aβ25-35 fragment exacerbated memory deficits, with enhanced caspase activation in the gene deletion mice compared to mice expressing FtMt (Wang et al., 2017). Such studies will be enhanced with a transgenic mouse line that overexpresses FtMt. In models of PD, increased expression of FtMt was shown in mice treated with the dopaminergic toxins 6-OHDA and MPTP, while similarly treated FtMt gene deletion mice had higher levels of dopaminergic cell loss (Shi et al., 2010; You et al., 2016).

To determine whether FtMt has potential as a therapeutic approach, possibly by gene delivery methods, there remains the question of how much FtMt is protective and if mitochondrial damage can occur if levels are too high. Our previous paper showed that overexpression of FtMt in the ARPE-13 line of retinol epithelium cells caused several effects on mitochondrial function including increased mitochondrial fission and mitophagy (Wang X. et al., 2016). In order to clarify these issues, we established neuronal cell lines with stable expression of high, medium and low FtMt levels. Using these cell lines, we examined effects of overexpression of FtMt on neuronal phenotype, neuroprotective activity, and gene expression profiles.

## Materials and Methods

### Cell Culture

The human neuroblastoma SH-SY5Y cell line was obtained from the American Type Culture Collection (Gaithersburg, MD, USA) (Ross et al., 1983). Cells were grown in Dulbecco's Modified Eagle's Medium (DMEM) with high glucose and supplemented with 5% fetal bovine serum (FBS) and 50 μg/ml gentamicin. All cell culture reagents were obtained from Nacalai-Tesque, Kyoto, Japan. Cells were routinely subcultured with 0.1% trypsin/1 mM EDTA in Hanks Balanced Salt Solution (HBSS) before reaching confluency, but in some circumstances during earlier stages of clone establishment, cells were subcultured with PBS without trypsin to separate the neuroblast from epithelial-type cells. After initial isolation of FtMt-expressing clones, all experiments were carried out with differentiated cells with the exception of studies on cell growth. To produce differentiated cells with neuronal features, untransfected and isolated clones were treated with retinoic acid (10 μM)(Sigma-Aldrich, St. Louis, MO, USA) for 7 days in DMEM with 0.5–1.0 % FBS. Media was refreshed after 3 days of culture.

### Transfection

Cells were transfected with a pEGFP-FTMT plasmid whose construction and characterization has been described (Wang et al., 2011). All recombinant DNA experiments were carried out with appropriate institutional approvals. To isolate stably-expressing SH-SY5Y cells, cells were plated at 2 × 10^5^ cells/well in 12 well-plates in growth media. After 24 h, media was replaced with serum-free DMEM, and transfection was carried out in triplicate using 0.5–1 μg plasmid DNA mixed with Viofectin (Viogen, New Taipei City, Taiwan) according to the manufacturer's instructions. After 6 h, serum was added to media to 5% final concentration, and cells allowed to recover for 18 h. The cultures were then subcultured with cells from each well-being transferred to a 60 mm diameter petri dish. The following day, media was replaced with growth media (DMEM + 5% FBS) containing 500 μg/ml G418 (Selection Media) (Nacalai-Tesque, Kyoto, Japan). The progress of the cultures were followed with twice-weekly media changes until growing colonies could be identified on plates. Individual colonies were selected using trypsin-soaked cloning discs (approximately 0.5 cm diameter), and these were transferred to individual T25 flasks in selection media for expansion. A number of separate colonies of G418-resistant cells were grown until cell numbers were sufficient to permit screening for FtMt expression and freezing in liquid nitrogen. A total of 26 isolated clones were isolated, screened and stored (Supplemental Figure 1).

### Western Blot Screening Colonies for FtMt Expression

Western blots were used to screen isolated clones for expression of FtMt protein in selected clones. Initial screening was carried out using undifferentiated cells. Cell pellets were briefly disrupted by sonication in RIPA buffer (50 mM Tris-HCl, pH 7.6, 1% sodium deoxycholate, 1% NP40, 0.1% sodium dodecyl sulfate (SDS) and a cocktail of protease inhibitors). Protein concentrations in samples were measured using a MicroBCA Protein assay kit (Thermo Scientific, USA). Samples were prepared for SDS-polyacrylamide gel electrophoresis by dissolving in SDS-sample buffer containing 0.1M dithiothreitol (DTT). Equal amounts of protein were loaded on precast 5–20% SDS gradient gels (Wako, Japan or Nacalai-Tesque, Japan). After separation, proteins were transferred to Immobilon P (Millipore, MA, USA) PVDF membrane using semi-dry electroblotting apparatus.

### Western Blot Detection and Quantification

The following procedures were used for all western blot in the analyses in this report. PVDF membranes were blocked for 1 h with 5% skim milk dissolved in Tris-buffered saline (50 mM Tris-HCl, pH 7.5, 150 mM NaCl) containing 0.05% Tween 20 (TBST). Membranes were subsequently incubated at room temperature for 18 h in optimal antibody dilution in 2% skim milk in TBST containing 0.005% sodium azide. The production and characterization of the custom rabbit polyclonal antibody to FtMt has previously been described (Yang et al., 2015). The antibodies used in this report are listed in Table 1. After antibody incubation, membranes were sequentially washed 3 × 10 min in TBST, and incubated in the appropriate horseradish peroxidase (HRP)-conjugated anti-immunoglobulin for 2 h. Membranes were imaged using Chemi-Lumi-One HRP chemiluminescence substrate (Nacalai-Tesque) with a LAS4000 Imaging system (GE Biosciences, U.S.A.). Some membranes were reprobed with different antibodies after treatment with Stripping Agent-Strong (Nacalai-Tesque). For normalization and loading control, membranes were subsequently incubated with an HRP-conjugated antibody to β-actin (FujiFilm/Wako, Japan) at 1:15,000 for 1 h. All images were quantified using Image Studio Lite software (Licor, NE, USA). Results are calculated as relative expression after normalization for levels of β-actin.

Table 1Description of reagents used in study.

**Table:** 

**Antigen**	**Supplier/Cat.no**	**Description**	**Species**	**Application**	**Dilution**
**ANTIBODIES**					
FtMt	Custom	Polyclonal	Rabbit	WB, ICC	1/5000
NFH/M	Biolegend (837904)	Monoclonals	Mouse	WB, ICC	1/2000
PSD95	Abcam (ab126732)	Monoclonal	Rabbit	WB	1/2000
SNAP25	Abcam (ab109105)	Monoclonal	Rabbit	WB	1/10000
APP	Abcam (ab126732)	Monoclonal	Rabbit	WB	1/3000
Tau	Pierce (MN1000)	Monoclonal	Mouse	WB	1/2000
α-synuclein	Covance 4B12	Monoclonal	Mouse	WB	1/2000
TXNIP	Abcam (ab188865)	Monoclonal	Rabbit	WB	1/2000
Mt-Cox-1	Abcam (ab14705)	Monoclonal	Mouse	WB, ICC	1/2000
Histone H3	Abcam (ab21054)	HRP Polyclonal	Rabbit	WB	1/10000
β-actin	Fujifilm (017-24573)	HRP Monoclonal	Mouse	WB	1/10000

*WB, Western blot; ICC, Immunocytochemistry*.

**Table:** 

**Gene**	**Sequence**	**Amplicon (bp)**	**Ref.Seq**.
**PCR PRIMER SEQUENCES**			
FTMT sense	CCCATTTGTGCGATTTCC	166	NM_177478.1
FTMT antisense	TTCTGCTTGTTTTCATTTCCA		
MGST-1 sense	GAAAGGTTTTTGCCAATCCA	186	XM_011520674
MGST-1 antisense	TTCCTGCCATTCTCATTTCC		
TXNIP sense	TCGTGTCAAAGCCGTTAGGA	228	NM_006472.5
TXNIP antisense	TTGAAGGATGTTCCCAGAGGC		
TXN sense	GCCTTTCTTTCATTCCCTCTCT	145	NM_003329.3
TXN antisense	ACCCACCTTTTGTCCCTTCT		
CALCA sense	GTCAAGGCACAGCATTACCA	95	XM_017018283.1
CALCA antisense	CCCTATTGACATTGGTGGCTCT		
SCG2 sense	AGATGAAACGCTCAGGGCAG	147	NM_003469.4
SCG2 antisense	GCCCATTCTGTAACCTCCCA		
GDF-15 sense	CTCTCAGATGCTCCTGGTGTT	159	AF003934.1
GDF-15 antisense	AGCAGGTCCTCGTAGCGTTT		
TFRC sense	GCCAATGTCACAAAACCAAA	188	NM_003234.3
TFRC antisense	AAGTCCTCTCCTGGCTCCTC		
β actin sense	CCTATGTGGGCGACGAG	242	NM_001101.3
β actin antisense	ATGGCTGGGGTGTTGAAG		

### Immunocytochemistry

Immunocytochemistry was used to demonstrate expression and localization of FtMt, neurofilaments, and mitochondrial cyclooxygenase 1 (MtCox-1) in differentiated FtMt expressing clones. Different clones were plated onto chamber slides (Nunc) at 10^4^ cells/well and differentiated for 7 days in RA-containing media. After that period, cells were rinsed with PBS, and fixed with 70% ethanol at −20°C for 30 min. The fixative solution was removed and cells dried to ensure attachment. Cells were incubated in optimal dilutions of antibody (Table 1) diluted in TBST overnight at room temperature. After washing three times with TBST, cells were incubated with optimal dilution of anti-species immunoglobulin conjugated with fluorophore (dyes used indicated in Figure legends). Cells were counterstained with DAPI to allow identification of nuclei and coverslipped with fluorescent mounting agent. Stained cells were examined using a FV1000 Olympus confocal microscope. Photos were taken from three randomly selected fields for each experiment of three independent experiments.

### Cellular Fractionation and Mitochondria Isolation

To measure the subcellular localization of overexpressed FtMt, differentiated cells were fractionated using the Mitochondria Isolation kit for Mammalian Cells (Thermo Fisher, USA) to isolate a mitochondria-enriched fraction. FtMt expressors and untransfected cells were grown in T75 flasks and differentiated for 7 days with RA treatment. Mitochondria fractions were isolated from collected cells following a modification of the manufacturer's recommended protocol. In brief, cell pellets containing approximately 1.5 x 10^7^ cells were resuspended in Reagent A (with added protease inhibitors) (400 μl) and incubated on ice for 2 min. 5 μl of Reagent B was then added and pellets were gently sonicated for 5 s to ensure complete cell lysis without damaging nuclei. The lysates were centrifuged for 10 min at 700 g and the supernatants transferred to new tubes. This step was repeated to remove all nuclear material. The supernatants were then centrifuged at 12,000 g for 15 min. The pellets were resuspended in 500 μl of Reagent C and centrifuged for the same period as a wash step, while the supernatants were saved as cytosolic fractions. The protein concentration was determined for each fraction, and samples processed for western blot analysis.

### Cell Replication and Neurite Elongation Measurements

Similar analysis methods were used to determine the rate of replication of undifferentiated FtMt clones compared to untransfected cells, and development of neurites of differentiated FtMt clones compared to untransfected cells. For measuring cell replication, 10^4^ cells from different clones were plated into T25 flasks in growth media. Phase contrast images were recorded at Day 1, Day 3, Day 5, and Day 7 using an Olympus phase contrast microscope. Area occupied was used as the measure of cell numbers. The area of cells in 20x images were measured using image J software (NIH, USA). Five images taken at random were recorded at each time point for each clone. Each experiment was repeated three times. Results recorded show mean values ± standard error of mean (SEM).

For measuring neurite formation, 10^4^ cells from each clone were plated into wells of 12-well plates in triplicate. The following day media was exchanged for differentiation media containing RA. Five images/well were recorded and analyzed for neurite elongation after 7 days using Image J software plugin NeuronJ (https://imagescience.org/meijering/). The area occupied by neurites in each field was measured and data analyzed as described above.

### Cell Viability Measurements

For assessing responses to oxidative stress caused by hydrogen peroxide or cobalt chloride, a microtiter assay was used. Using 96-well-tissue culture microtiter plates, 1.5 × 10^4^ cells/well were plated in 100 μl of media. After attachment, media was exchanged for differentiation media and cells were treated for 7 days to develop a neuronal phenotype. For assessing treatments, media was exchanged for DMEM + 1% FBS to which different concentrations of hydrogen peroxide (0–250 μM) or cobalt chloride (0–200 μM) were added. Cells were incubated for 24 h and then 10 μl of cell viability reagent WST-1 (Cell Count Reagent, Nacalai-Tesque, Japan) was added to each well. Absorbance at 450 nm was recorded using a Tecan 2000 plate reader after 1, 2 and 4 h of further incubation. Changes in absorbance were calculated after subtraction of absorbance from cell-free wells. Degree of protection was calculated as percentage changes relative to untreated cells for each clone.

### RNA Preparation

RNA was prepared from 7 day RA-differentiated neuronal-like cells (FtMt clones and untransfected cells) using the RNeasy plus Mini kit (Qiagen, Hilden, Germany) according to the manufacturer's instructions. RNA yield was assessed using a Nanodrop spectrophotometer (Thermo Scientific, USA), and integrity with an Agilent Bioanalyzer and RNA 6000 Nano kit (Agilent Inc, Palo Alto, USA). All RNA samples had RIN values >9 and were considered suitable for microarray analysis.

### Microarray Analysis

Microarray analysis using the Applied Biosystems Human Genome U133 Plus 2.0 array (ThermoFisher Scientific, USA) was carried out as a contract analyses by Takara Bio Inc. (Kusatsu, Japan) using RNA isolated from the clone with the highest expression of FtMt, the medium expression FtMt clone, and untransfected control cells. Labeled cRNA probes from RNA were prepared using the 3' IVT Plus transcription kit (Thermo Fisher Scientific). The array contained 11 separate oligonucleotide probes to represent each of over 47,000 transcripts. Data was extracted and analyzed using Affymetrix Signal Console software. Signal intensity for each gene oligonucleotide probe was obtained based on the mean of oligonucleotide replicates after correction for normalization controls.

### Microarray Data Analysis

Results of analysis of gene expression were prepared by pairwise comparison of untransfected (negative) to high FtMt expression; untransfected (negative) to medium FtMt expressor, and medium to high FtMt expressors. Intensity data for each clone for each gene probe was log_2_ transformed. Significant differences between gene expression values were highlighted when the difference in log_2_ values varied by +1 or −1 (reflecting 2.7 fold increase or decrease in expression). Adjusted *p*-values were provided that reflect significant differences in expression based on the 11 oligonucleotide/transcript technical replicates. Tables were prepared that highlighted significant differences in gene expression, but excluding low expressing genes marked as Absent (below background), and genes with no assigned identity. Different processes of data analysis were examined to demonstrate how altered levels of FtMt affect cellular phenotypes. Particular emphasis was placed on data mining for genes important for protection from oxidative stress, iron transport, and those involved in neuronal differentiation and phenotypes.

### Real Time Reverse Transcription-Polymerase Chain Reaction

Quantification of FtMt gene expression and validation of identified genes with differential expression was carried out by real time RT-PCR with SYBR green detection using RNA from the high and medium FtMt overexpressing clones used for microarray analysis and untransfected cells, and also one additional high expressor and one low FtMt expressor clone. RNA was isolated as described above, and equal amount of RNA (0.75–1 μg total cellular RNA) from each sample was reverse transcribed using the PrimeScript RT kit with genomic DNA eraser (Takara Bio, Japan). Appropriate numbers of samples that were not reverse transcribed were analyzed to verify that amplification signal was not due to contaminating DNA.

Real time PCR was carried out using a relative standard curve method (Walker et al., 2009) for analysis using Thunderbird SYBR QPCR master mix supplemented with Rox reference dye (Toyobo, Inc, Japan). Primers (listed in Table 1) were used at 12.5 pmol/reaction, and each sample was analyzed in duplicate using triplicate biological replicates on the Roche Light Cycler 480 qPCR machine using the following program: 95°C 60 s denaturation/activation, followed by amplification program of 95°C for 15 s, 60°C for 20 s and 72°C for 30 s for 45 cycles. PCR experimental design followed most of the criteria for Minimum Information for Publication of Quantitative Real time PCR Experiments (MIQE) (Bustin et al., 2009). Primers were designed and analyzed to show absence of self-dimers or cross dimers, and this was verified by demonstration of melting curves that showed single bands.

### Statistical Analysis

Statistical analyses were carried out using Graphpad Prism 7 software (La Jolla, CA). Analysis between groups and treatments were carried out by One-way or Two-Way Analysis of Variance (ANOVA) with Tukey's test of multiple comparisons to examine significance between groups. Significance was assessed if *p* < 0.05.

## Results

### Isolation of Mitochondrial Ferritin Overexpressing SH-SY5Y Cells

Using standard cell transfection methodology and a plasmid that expressed the human FtMt gene using the CMV immediate early gene promoter, we isolated by disc cloning 26 separate G418-resistant colonies and expanded them in separate cultures. These were screened for expression of FtMt protein by western blot (Supplemental Figure 1). As this image was obtained during the screening process, it used protein extracts from undifferentiated cells. As shown, there was a wide range of FtMt expression levels ranging from undetectable to high levels of expression. Isolated clones were expanded and then frozen, and for subsequent experiments, each clone was used for 6–8 passages before a new frozen vial was revived. Expression and growth of some of the highest expressors proved unstable, but final characterization was carried out on 4 separate clones as their growth and different FtMt expression levels were stable with passage.

**Figure 1:**
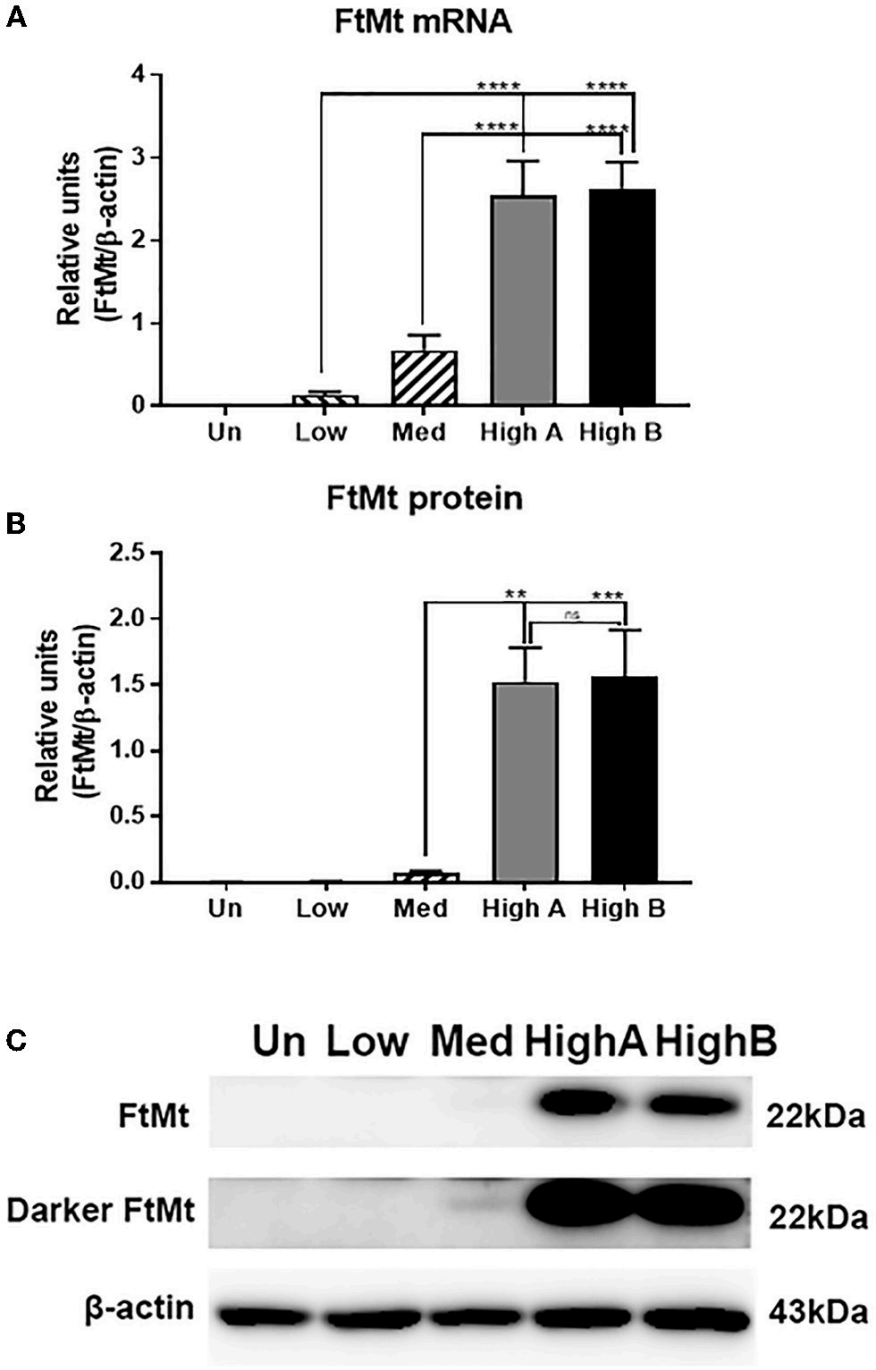
**(A)** FtMt mRNA expression in FtMt expressing clones and untransfected differentiated SH-SY5Y cells: Real time PCR results showing comparison of FtMt mRNA levels in indicated clones compared to untransfected cells normalized for levels of β-actin mRNA. Results show combination of triplicate independent experiments with 2 or 3 samples/experiment. Data analyzed by One-way ANOVA with Tukey's *post-hoc* comparison test. Bar charts illustrate mean ± standard error of mean (SEM). Results with *p-*values of significance are indicated. *p < 0.05; **p < 0.01, ***p < 0.001, ****p < 0.0001. **(B)** FtMt protein expression in FtMt expressing clones and untransfected differentiated SH-SY5Y cells: Western blot results showing comparison of FtMt protein levels in indicated clones compared to untransfected cells normalized for levels of β-actin protein. Results show combination of triplicate independent experiments with 2 or 3 samples/experiment. Data analyzed by One-way ANOVA with Tukey's *post-hoc* comparison test. Bar charts illustrate mean ± SEM. Results with *p*-values of significance are indicated. *p < 0.05; **p < 0.01, ***p < 0.001, ****p < 0.0001. **(C)** Representative Western blots of FtMt expressing clones and untransfected differentiated SH-SY5Y cells: Representative western blot image of data shown in **(C)**. FtMt expression was highly expressed in clones High A and High B. A longer exposure was required to show FtMt protein in Medium (Med) expressing clone (Darker FtMt).

Relative levels of FtMt mRNA and protein were measured by real time polymerase chain reaction (qPCR) and western blot. Two clones had high levels of expression of FtMt mRNA and protein (Figures 1A,B), while one had medium level of FtMt mRNA expression and low levels of detectable protein, and one had low level of FtMt mRNA expression but with no detectable protein (representative western blot: Figure 1C). The difference in mRNA expression between the low and high clones was 20-fold, and 4-fold between medium and high, but for protein levels, the difference in expression between medium and high was 22-fold. Considering the differences in mRNA expression, it might be expected that FtMt protein should be detectable in the low expressor clone. These differences between mRNA and protein would suggest that FtMt protein was being rapidly degraded. It should be noted that untransfected SH-SY5Y differentiated cells used in these experiments did not contain detectable levels of FtMt mRNA or protein.

### Phenotyping of FtMt Overexpressing Clones

#### Characterization of FtMt Expressing Clones

**Figure 2:**
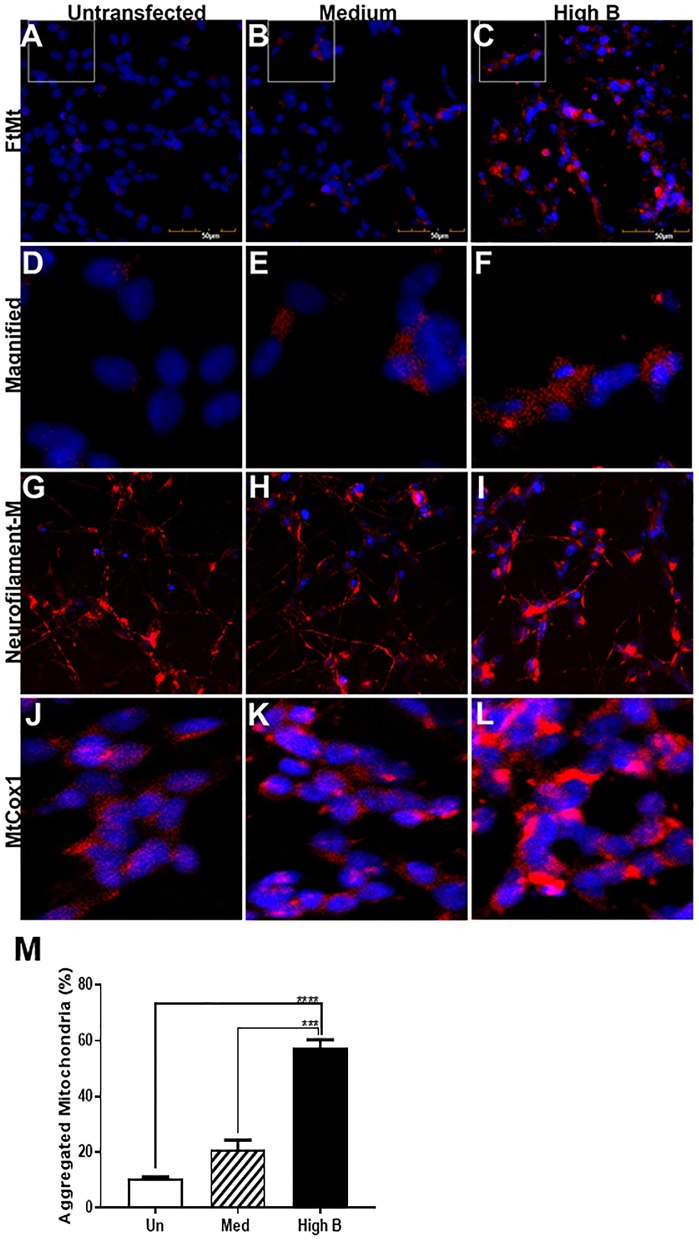
Immunocytochemistry of FtMt expressing clones. **(A–C)**. Untransfected **(A)**, Medium **(B)**, and High B **(C)** clones differentiated for 7 days stained with antibody to FtMt to identify positive expressing cells. Positive staining is observed for Medium and High B clone, but not untransfected cells. Antibody reacted cells were identified by reaction with Alexa568 labeled-anti rabbit immunoglobulin G. All images were recorded using an Olympus FV1000 laser confocal microscope. **(D-F)**. Higher magnification images of FtMt antibody reacted cells. Images from areas indicated with white lines on **(A–C)**. **(G–I)** Untransfected **(G)**, Medium **(H)**, and High B **(I)** clones differentiated for 7 days stained with antibody SMI-312 that stains neurofilament proteins, but predominantly NF-M. All cells showed SMI-312 positive neuronal processes consistent with differentiated morphology. Undifferentiated cells show no staining for SMI312 (not shown). Antibody-reacted cells were identified by reaction with Alexa568 labeled-anti mouse immunoglobulin **(G)**. **(J–L)**. Untransfected **(J)**, Medium **(K)**, and High B **(L)** clones differentiated for 7 days stained with antibody MtCox1 that stains mitochondrial cytochrome oxidase-1. Morphology of MtCox1 immunoreactive mitochondria in FtMt expressing clones (**K**-Medium and **L**- High B) compared to untransfected cells **(J)**. Antibody reacted cells were identified by reaction with Alexa568-labeled anti mouse immunoglobulin **(G)**. **(M)** Quantitative estimation of aggregated/abnormal mitochondria. Estimation of percentage of aggregated mitochondria in untransfected (Un), Med, and High B expressing clones. The numbers were estimated by visual examination of 3 fields/image and 3 images for each clone at magnification shown in **(A–C)**. Results show mean ± SEM, with standard significance levels indicated from data analyzed by one way ANOVA with Tukey *post-hoc* test. ***p < 0.001, ****p < 0.0001.

As elevated amounts of FtMt will increase the levels of potentially toxic iron in mitochondria, a series of experiments were carried out to determine how overexpression affected the phenotype and gene expression of clones differentiated to a neuronal phenotype. Staining of cells with antibody to FtMt showed expected clear differences in immunoreactivity intensity between medium and high expressor cells (Figures 2B,C), while the untransfected cells only showed some background staining (Figure 2A). Higher magnification images are shown in Figures 2D–E. All cells of the high expressor clone showed FtMt immunoreactivity (Figures 2C,F). The punctate immunoreactivities are consistent with mitochondrial localization. Staining of cells with pan-neurofilament antibody (SMI312) showed that all cells produced the characteristic processes of neurons (Figures 2G–I). Staining of cultures with an antibody to mitochondrial cytochrome oxidase1 (MtCox-1) showed an unexpected gradation of distribution and intensity of mitochondria in the untransfected, medium expressor and high expressor cells (Figures 2J–L). Many of the high FtMt expressing cells showed MtCox1 staining consistent with an aggregated morphology. This was confirmed by determining the percentage of cells with aggregated morphology of MtCox-1 immunoreactivity. This showed significant increase between the untransfected and high expressing clone (Figure 2M).

#### Subcellular Localization of Mitochondrial Ferritin

**Figure 3:**
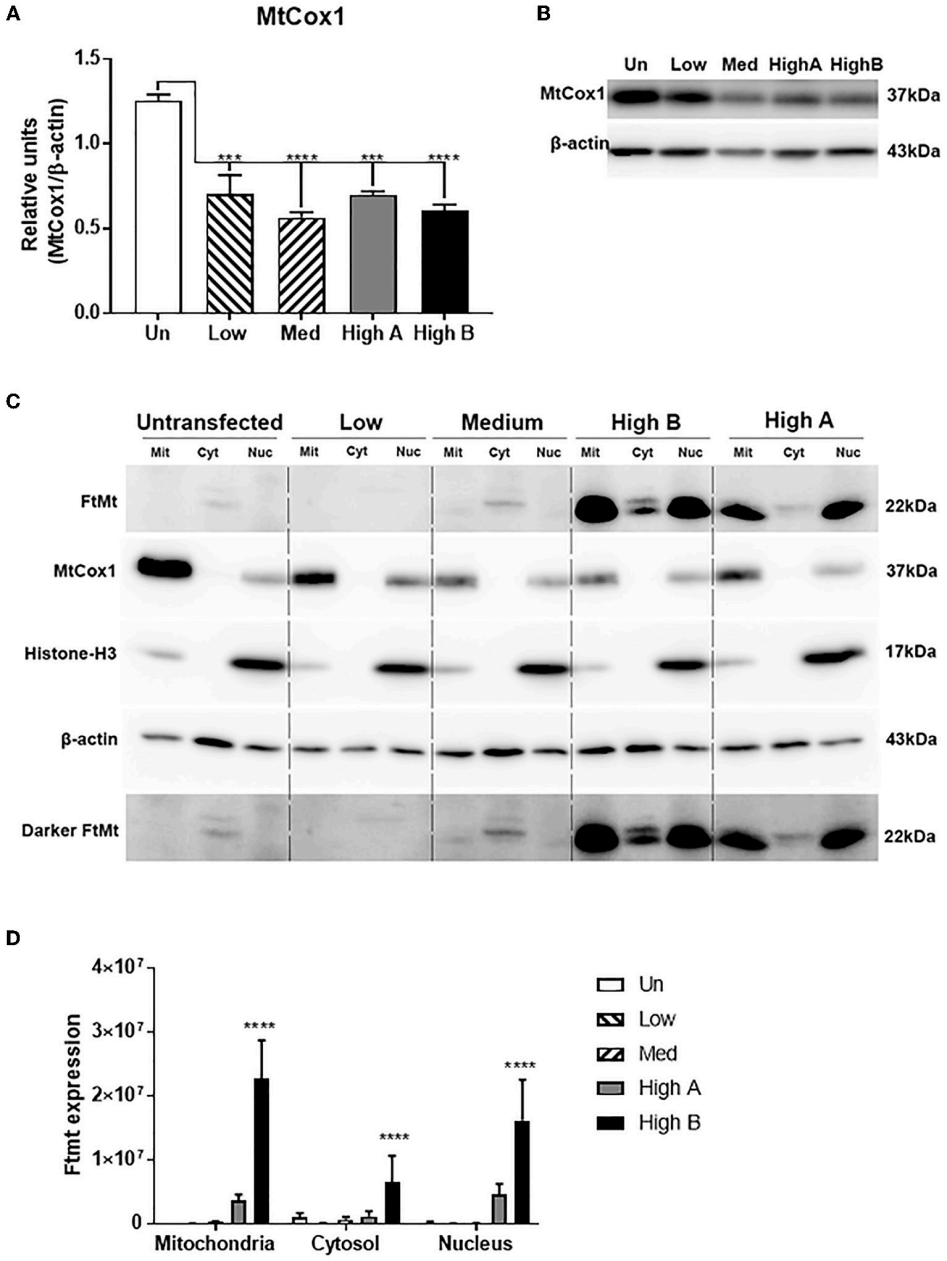
Characterization of mitochondria in FtMt expressing clones. **(A,B)** Western blot measurements of MtCox-1 in whole cell extracts. Measurement of Mt Cox-1 in total cell lysates by western blots gave the reverse result compared to immunocytochemistry. There were significantly reduced levels of MtCox-1 protein in FtMt expressing clones. Results based on triplicate determinations. The different patterns of expression are shown in representative western blot. **(C)** Composite western blot panel of subcellular fractions from FtMt expressing clones. Cell extracts from differentiated clones fractionated into mitochondria (Mit), cytosol (Cyt), and nucleus (Nuc) fractions were analyzed by western blot and probed with antibodies to FtMt, MtCox1, Histone -H3, and β-actin. The results for MtCox1 and Histone-H3 indicate that the mitochondria contain some contaminating nuclei, and the nuclei contain some contaminating mitochondria. The results show that the majority of overexpressed FtMt is present in mitochondria though the abundance in nucleus is higher than might be expected from mitochondria contamination. Enhanced exposure was needed to demonstrate FtMt in Med clone (Darker Ftmt). A non-specific band of higher molecular weight than FtMt. is detected in cytosol fraction. **(D)** Quantitative estimation of results in **(A)**. Results show relative levels of FtMt protein in different fractions from FtMt expressing clones. Results are not normalized and represent signal intensities from triplicate experiments. Results show mean ± SEM, with standard significance levels indicated from data analyzed by one way ANOVA with Tukey's *post-hoc* test. ***p < 0.001, ****p < 0.0001.

Quantification of MtCox-1 expression by western blots of total cell lysates detected the opposite pattern with significantly higher levels in untransfected neurons (Figures 3A,B). The reason for this discrepancy requires further investigation, but both data suggest abnormalities of mitochondria.

The levels of FtMt protein in different cellular fractions, particularly mitochondria, were measured by western blot. The method used produced a mitochondria-enriched fraction, and also a nuclear and cytosolic fraction, from each cell extract. The question being asked was whether the considerably increased amounts of FtMt being produced by overexpressing cells accumulated predominantly in the mitochondria fraction. The western blots were representative of distinct experiments carried out in triplicate (Figure 3C). The results showed that FtMt was predominantly detected in the mitochondria fraction, but a certain amount could also be detected in the nuclear fraction of the highest expressing clone (Figure 3C - HighB). The levels of FtMt in the nuclear fraction of this clone were higher than might be expected from contamination of this fraction by mitochondria. In the cytosolic fraction, we detected two polypeptide bands of slightly higher molecular weight than processed FtMt. As these were also detected in the untransfected cells that lack FtMt RNA, these bands were considered as non-specific. Similar to Figure 3B, the western blots showing levels of MtCox1 in the different fractions showed larger amounts of this protein in the untransfected cells. The relative intensities of FtMt in the different fractions are illustrated in Figure 3D.

#### Alteration in Expression of Neuronal Proteins

**Figure 4:**
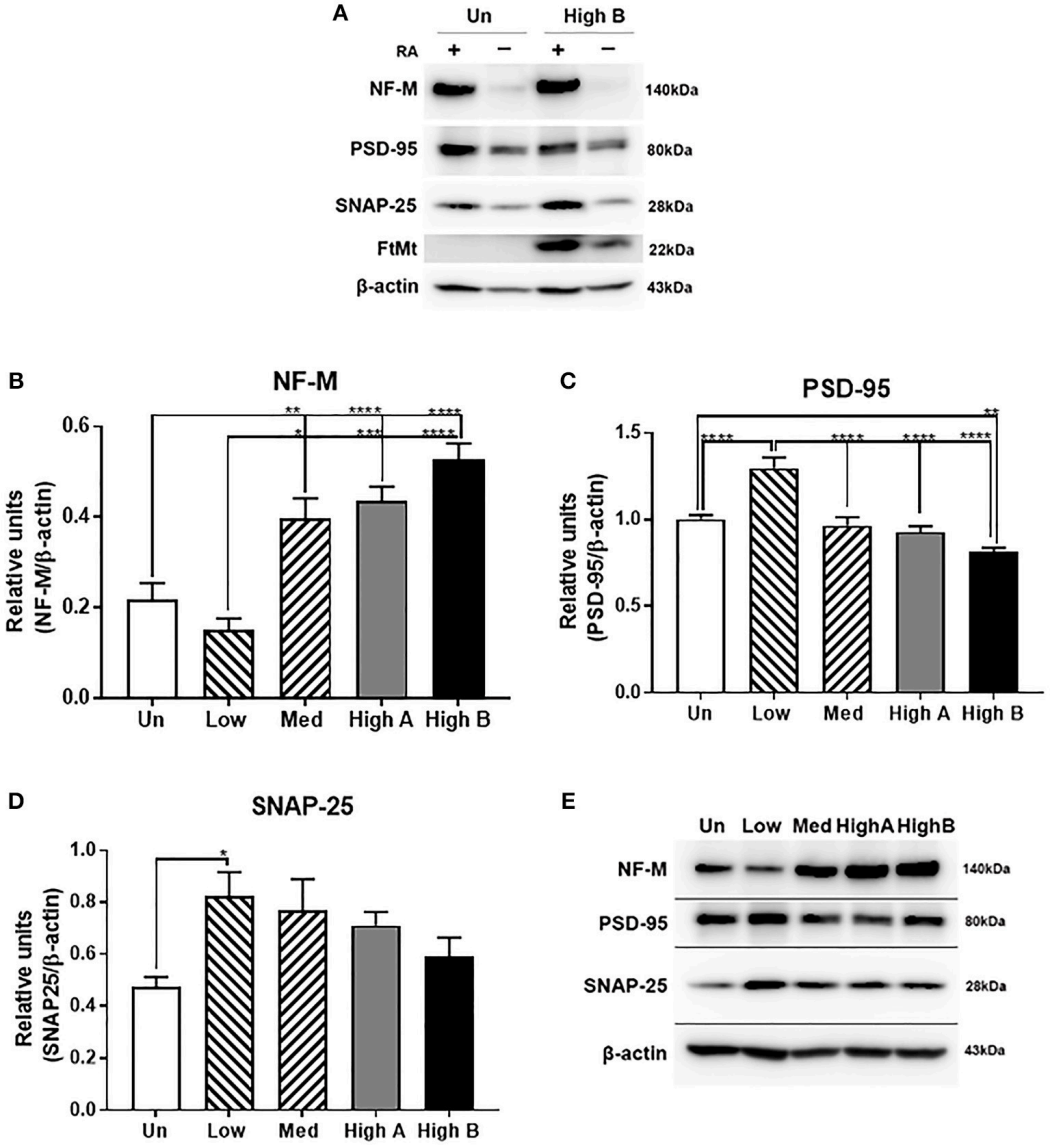
Expression of neuronal proteins in FtMt clones. **(A)** Composite picture of western blots showing the differences in levels of NF-M, PSD-95, SNAP-25, and FtMt between undifferentiated (-) and RA differentiated (+) of untransfected (Un) and FtMt high expressing clone (High B). It was noticed in all experiments that FtMt expression was increased with differentiation RA treatment, even though the expression vector was using the CMV immediate-early promoter, and not the native FtMt promoter. **(B)** Relative levels of NF-M **(B)** in different FtMt clones. Results shown in B represent mean ± SEM of triplicate experiments. Data analyzed by One way ANOVA with Tukey's *post hoc* test of significance. Results indicate level of significance. *p < 0.05; **p < 0.01, ***p < 0.001, ****p < 0.0001. **(C)** Relative levels of PSD-95 **(C)** in different FtMt clones. Results shown in C represent mean ± SEM of triplicate experiments. Data analyzed by One way ANOVA with Tukey's *post-hoc* test of significance. Results indicate level of significance. *p < 0.05; **p < 0.01, ***p < 0.001, ****p < 0.0001. **(D)** Relative levels of SNAP-25 **(D)** expression in different FtMt clones. Results shown in -D represent mean ± SEM of triplicate experiments. Data analyzed by One way ANOVA with Tukey's *post-hoc* test of significance. Results indicate level of significance. *p < 0.05. **(E)** Composite Western blot showing pattern of expression for NF-M, PSD-95, and SNAP-25. Representative western blots of one experiment showing patterns of expression in untransfected and different FtMt clones. Experiment represents the sequential probing of single membrane for NF-M, PSD-95, and SNAP-25 followed by normalization for β-actin.

To determine if FtMt overexpression affects neuronal phenotype upon differentiation, expression levels of key neuronal proteins-neurofilament M (NF-M); synapse-associated proteins, postsynaptic density protein 95 (PSD95) and synaptosomal-associated protein-25 (SNAP-25)- were measured by western blotting (Figure 4). To confirm the effect of retinoic acid differentiation on expression of proteins, differentiated, and undifferentiated cells from untransfected and High B FtMt clone were analyzed for NF-M, PSD-95, SNAP-25 and FtMt (Figure 4A), which shows that these neuronal proteins were upregulated by RA differentiation. Surprisingly, it was also observed that there was increased amounts of FtMt in the overexpressing clone following RA treatment. This was unexpected as FtMt in these clones was being expressed using the plasmid CMV IE promoter not the native FtMt promoter.

There were significantly higher levels of NF-M in higher FtMt overexpressing clones compared to the untransfected and low expressor (Figure 4B). By comparison the low expressor had significantly higher levels of the synaptic proteins PSD-95 (Figure 4C) and SNAP25 (Figure 4D). The pattern of expression for both proteins between the different cell types analyzed was similar with decreasing expression from low to high. A composite western blot showing these three proteins along with β-actin normalization for one representative experiment is shown (Figure 4E). NF-M, PSD-95, SNAP-25, and β-actin were sequentially detected on the same membrane. Although we could not detect FtMt protein by western blot in the analyses made in the low FtMt expressing clone, we did show significant expression of FtMt mRNA in these cells compared to untransfected cells, and assume that small amounts of FtMt protein are functional in this clone, but the turnover of this protein makes it undetectable. Further investigations are needed to improve sensitivity of detection, but these results and subsequent data suggest this low FtMt expressing clone has significant differences from untransfected cells.

#### Alteration in Expression of Amyloid Precursor Protein, Tau, and α-synuclein

**Figure 5:**
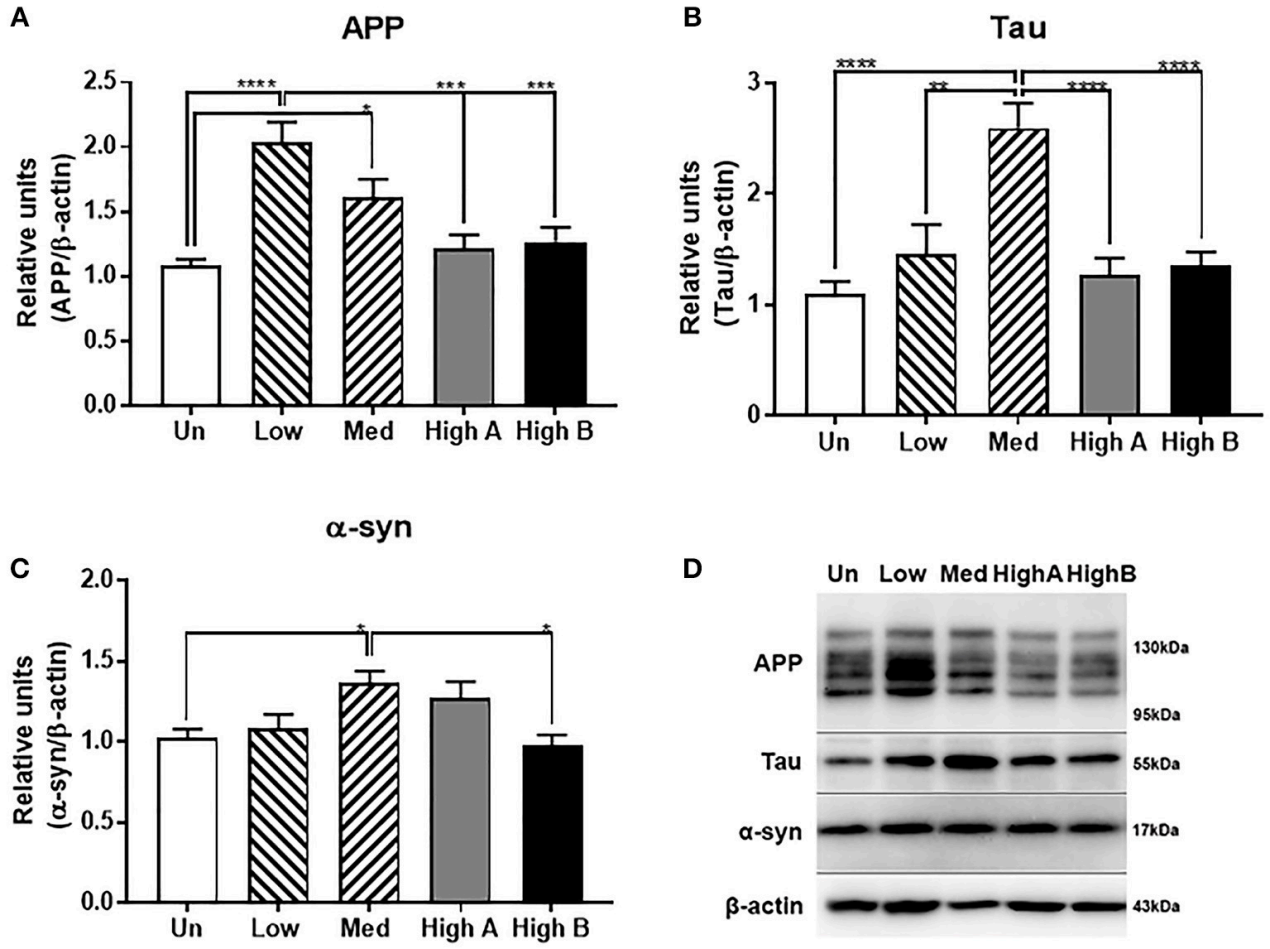
Expression of neuronal disease-associated proteins in FtMt clones. **(A)** Relative levels of amyloid precursor protein (APP) in different FtMt clones. Results shown represent mean ± SEM of triplicate experiments. Data analyzed by One way ANOVA with Tukey's *post-hoc* test of significance. Results indicate level of significance. *p < 0.05; **p < 0.01, ***p < 0.001, ****p < 0.0001. **(B)** Relative levels of Tau in different FtMt clones. Results shown represent mean ± SEM of triplicate experiments. Data analyzed by One way ANOVA with Tukey's *post-hoc* test of significance. Results indicate level of significance. *p < 0.05; **p < 0.01, ***p < 0.001, ****p < 0.0001. **(C)**. Relative levels of α-synuclein (α-syn) in different FtMt clones. Results shown represent mean ± SEM of triplicate experiments. Data analyzed by One way ANOVA with Tukey's *post-hoc* test of significance. Results indicate level of significance. *p < 0.05. **(D)** Composite Western blot showing pattern of expression for APP, tau, and α-synuclein. Representative western blots of one experiment showing patterns of expression in untransfected and different FtMt clones. Experiment represents the sequential probing of single membrane for APP, tau, and α-synuclein followed by normalization for β-actin.

Similar analyses were carried out for expression of amyloid precursor protein (APP) and microtubule associated protein tau, proteins associated with AD pathology, and α-synuclein, which is associated with PD (Figure 5). Expression of APP was generally higher in FtMt-expressing clones with highest levels of expression in the lowest expressing clone (Figure 5A). By contrast, a different pattern was seen for tau, with significantly increased expression in the medium FtMt expressor (Figure 5B). Highest α-synuclein expression was also observed in this clone, but the magnitude of change was less than for tau (Figure 5C). A composite western blot showing these three proteins along with β-actin normalization for one representative experiment is shown (Figure 5D). APP, tau, α-synuclein and β-actin were sequentially detected on the same membrane. The pattern of expression for tau was also observed in the gene expression analyses of these clones (see **Table 7**—MAPT).

### Effect of FtMt Overexpression on Neuronal Phenotypes

#### Cell Division and Neurite Formation

**Figure 6:**
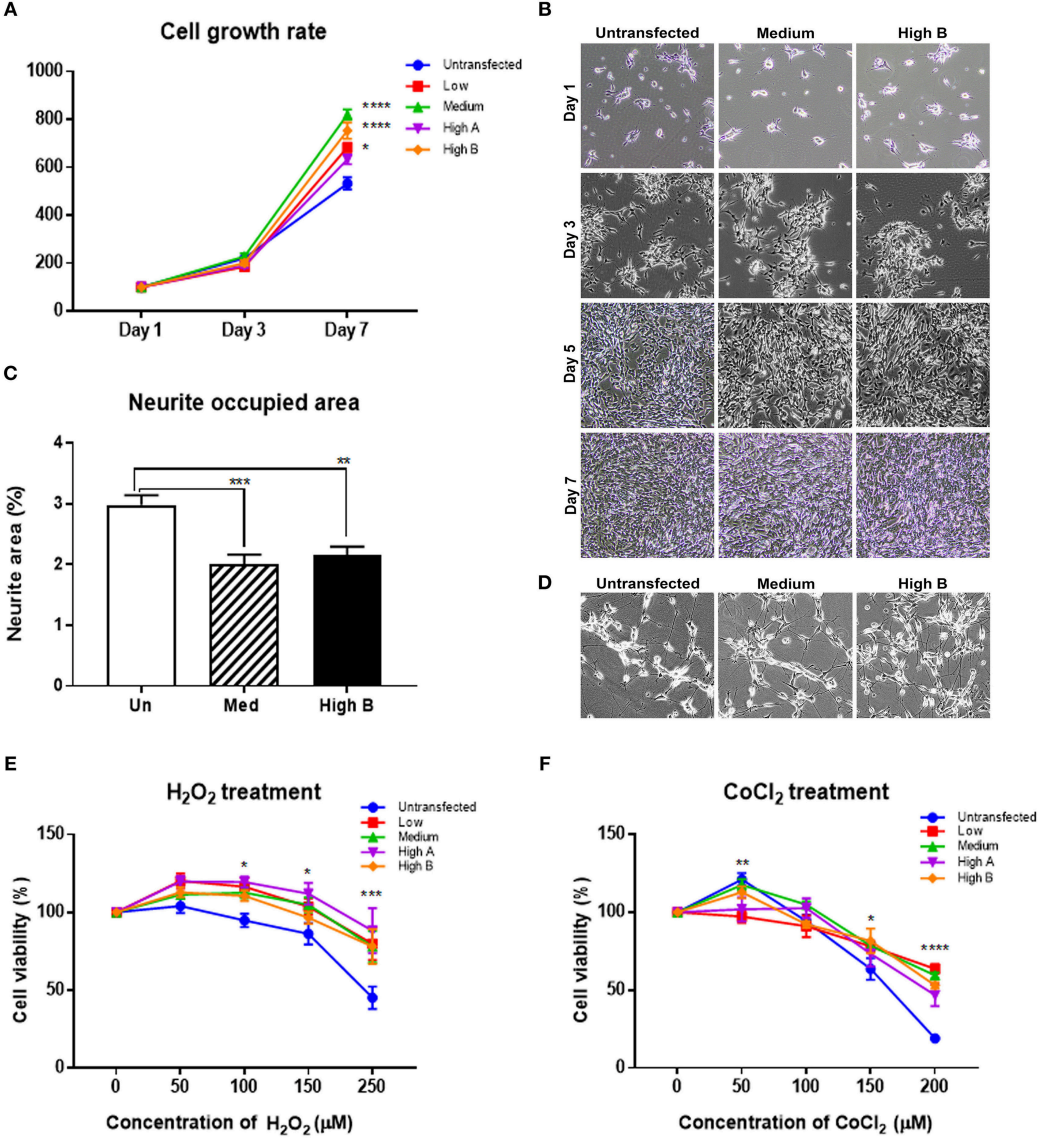
Phenotyping of FtMt expressing neuronal cells. **(A,B)** Measurements of Cell Growth Rates of Undifferentiated FtMt clones. **(A)** Results showing relative increase in cell area occupied as percentage of Day 1 of FtMt clones and untransfected cells. Results show analyses at Day 7. Measurements involved area occupied of growing cells identified using Image J image analysis software. Data analyzed by Two-way ANOVA. Relative statistical differences between clones and are listed in Table 2A. **(B)** Representative photomicrographs of proliferating clones untransfected, Medium FtMt expressor and High B FtMt expressor at Day 1, Day 3, Day 5, and Day 7. **(C,D)** Measurements of Neurite formation of Differentiated FtMt clones. **(C)** Results showing relative area occupied of neuritic processes at day 7 of untransfected (Un), Medium FtMt expressor (Med) and High Ft expressor (High B). Measurements involved measuring area occupied of neurites identified using NeuronJ plugin of Image J image analysis software. Results are representative of triplicate experiments. Relative statistical differences between clones listed in Table 2B. **(D)** Representative photomicrographs of differentiated, untransfected, Medium FtMt expressor and High B FtMt expressor at Day 7. **(E,F)** Responses of FtMt overexpressing clones to oxidative stress. **(E)** Changes in cell viability (as percentage of untreated cultures) with increasing doses of hydrogen peroxide (H_2_O_2_) (0–250 μM). Untransfected and FtMt expressing clones were differentiated in microtiter plate wells for 7 days. Treatments added to media lacking RA containing 1% FBS on day 7 for 24 h. Cell viability assessed by added WST-1 reagent after 24 h. Absorbance (450 nm) measured after 1, 2, and 4 h. Results presented (mean ± SEM) of six wells/treatment and combination of three independent experiments. Data analyzed by Two Way ANOVA. Relative statistical differences between clones and treatments are listed in Table 3A. **(F)** Changes in cell viability (as percentage of untreated cultures) with increasing doses of Cobalt Chloride (CoCl_2_) (0–200 μM). Untransfected and FtMt expressing clones were differentiated in microtiter plate wells for 7 days. Treatments added to media lacking RA containing 1% FBS on day 7 for 24 h. Cell viability assessed by added WST-1 reagent after 24 h. Absorbance (450 nm) measured after 1, 2, and 4 h. Results presented (mean ± SEM) of six wells/treatment and combination of three independent experiments. Data analyzed by Two-way ANOVA. Results indicate level of significance. *p < 0.05; **p < 0.01, ***p < 0.001, ****p < 0.0001. Relative statistical differences between clones and treatments are listed in Table 3B.

Comparison was made between undifferentiated clones of their rate of growth (Figure 6A) (Table 2A) and neurite formation (Figure 6C) (Table 2B). Representative photomicrographs for these experiments are shown in Figures 6B,D. These analyses showed that FtMt expressing clones replicated at a slightly faster rate over 7 days than the untransfected parent cells. By comparison, the medium expressor and high expressor clones produced significantly less density of neurites after retinoic acid differentiation for 7 days.

**Table 2:** Effect of FtMt overexpression on Cell division and differentiation.

**Sample**	***P*-values**
**(A) EFFECT OF FtMt ON CELL REPLICATION OF ISOLATED CLONES**	
Untransfected vs. Low	0.026
Untransfected vs. Medium	<0.0001
Untransfected vs. High A	0.3530
Untransfected vs. High B	<0.0001
**(B) EFFECT OF FtMt OVEREXPRESSION ON NEURITE FORMATION OF**	
**DIFFERENTIATED CELLS**	
Untransfected vs. Medium	0.0002
Untransfected vs. High B	0.0013
Medium vs. High B	0.7856

*Summary of results at day 7 between clones as illustrated in Figure 6B. Areas occupied by non-differentiated cells were measured using ImageJ from 5 separate random fields of each cell type. Results analyzed by Two Way Analysis of Variance with Tukey's post-hoc test. Results are compiled from three separate experiments*.

*Summary of results at day 7 between clones as illustrated in Figure 6D. Areas occupied by neurites formed from retinoic acid differentiated cells were measured using ImageJ with NeuronJ plugin from 5 separate random fields of each cell type. Results analyzed by Two Way Analysis of Variance with Tukey's post-hoc test. Results are compiled from three separate experiments*.

#### Resistance to Oxidative Stress From Hydrogen Peroxide or Cobalt Chloride

Cytotoxicity assays were carried out using hydrogen peroxide and cobalt chloride as inducers of oxidative stress to determine if the FtMt expressing clones showed more resistance to these agents. These agents induce oxidative stress through different mechanisms, with hydrogen peroxide being a direct source of reactive oxygen species and cobalt chloride modeling oxidative stress caused by hypoxia (Yu and Gao, 2013). FtMt expressing clones appeared significantly more resistant to the toxic effects of hydrogen peroxide compared to untransfected cells (Figure 6E). Summary of statistical pairwise comparison for different doses of hydrogen peroxide is shown in Table 3A. These results confirm an earlier observation that differentiated cells are more resistant to different oxidative stress inducing factors (Cecchi et al., 2008; Cheung et al., 2009; Lee et al., 2015; Forster et al., 2016). A similar pattern of resistance was shown to cobalt chloride with significant protection in FtMt overexpressing clone (Figure 6F). Summary of statistical pairwise comparison for different doses of cobalt chloride is shown in Table 3B.

**Table 3:** Effect of FtMt overexpression on resistance to oxidative stress by hydrogen peroxide and cobalt chloride.

**Samples**	**Significance (*****P*****-values)**				
**Dose (μM) HydrogenPeroxide**	**0**	**50**	**100**	**150**	**250**
Untransfected vs. Low	0.99 (NS)	0.15 (NS)	0.028	0.106 (NS)	0.0001
Untransfected vs. Medium	0.99 (NS)	0.80 (NS)	0.1 (NS)	0.086 (NS)	0.0004
Untransfected vs. High A	0.99 (NS)	0.22 (NS)	0.019	0.013	0.0001
Untransfected vs. High B	0.99 (NS)	0.66 (NS)	0.16	0.52 (NS)	0.0003
**Dose (μM) Cobalt Chloride**	**0**	**50**	**100**	**150**	**200**
Untransfected vs. Low	0.99 (NS)	0.0005	0.98 (NS)	0.058 (NS)	0.0001
Untransfected vs. Medium	0.99 (NS)	0.94 (NS)	0.4 (NS)	0.048	0.0001
Untransfected vs. High A	0.99 (NS)	0.006	0.61 (NS)	0.30 (NS)	0.0001
Untransfected vs. High B	0.99 (NS)	0.46 (NS)	0.99 (NS)	0.01(NS)	0.0001

*Summary of results between clones as illustrated in Figure 6E (hydrogen peroxide) and Figure 6F (cobalt chloride) after 24 h treatment. Results analyzed by Two-Way Analysis of Variance with Tukey's post-hoc test. Results are compiled from three separate experiments. NS, not significant statistically*.

#### Gene Expression Profiling of FtMt Overexpressing Neuronal Cells

Gene expression profiling using microarrays was carried out with RNA derived from High (B) expressor, medium expressor and untransfected RA-differentiated neurons. Complete results are provided in a searchable Microsoft Excel file (Supplemental File 1) that shows all expression intensities of indicated genes for these clones. For analysis of differential gene expression, expression intensities were log_2_ transformed and differences of 1 log_2_ or greater (more than 2.7 fold) were considered of significance. The microarray platform used for these analyses did not include probes for FtMt, so we cannot directly compare gene expression values with levels of FtMt expression.

Initial inspection of this large dataset revealed that expression of many genes were marked as absent (below the level of detection). It was decided not to consider results from any genes where one or more of the values were tagged as absent. Another noticeable feature was that a number of genes had multiple results that were attributed to different Genbank accession numbers. This indicated different results depending on the gene sequence used to design the oligonucleotide probes on the microarray. Our analysis only considered the gene set with the highest expression values as low expression values tended to be highly variable and therefore less reliable. Finally, we excluded from analyses unassigned or uncharacterized genes even though some showed significant differential expression. Expression of commonly used housekeeping genes (ACTB, GAPDH, TUBB, PPIA, HPRT1, G6PD, B2M) showed no significant differences (<1 log_2_ difference) between the different analyzed cells.

Table 4 presents the top 20 upregulated and downregulated genes selected in order between the high FtMt expressor and untransfected neurons (left columns) and in adjacent columns, the differences between medium expressor and untransfected (middle columns) and between high and medium expressors (right columns). A complete list is presented in Supplemental Data that lists all genes with differential expression of >1 log_2_ (Supplemental File 2). Table 5 presents the top 20 upregulated and downregulated genes selected in order between the medium FtMt expressor and untransfected neurons (left columns) and in adjacent columns, the differences between high expressor and untransfected (middle columns) and between high and medium expressors. Table 6 presents the top 20 upregulated and downregulated genes selected in order between the high FtMt expressor and medium FtMt expressor (left columns), and in adjacent columns, the differences between high expressor and untransfected cells (middle columns) and between medium and untransfected cells (right column). Most of these genes followed the pattern of highest or lowest expression in high FtMt expressor, followed by moderate FtMt expressor, followed by untransfected cells (Complete gene lists for Table 5, 6 are included in Supplemental Files 3, 4).

**Table 4:** Differential Genes expressed by FtMt overexpressing SH-SY5Y neuronal cells.

**GeneSymbol**	**RefSeq**		**Log2**	**Fold**	**Log2**	**Fold**	**Log2**	**Fold**
			**Diff**	**Change**	**Diff**	**Change**	**Diff**	**Change**
			***HighB vs. Un***		***Med vs. Un***		***HighB vs. Med***	
**UPREGULATED GENES**								
GDF15	AF003934		4.39	21.02	1.85	3.60	2.55	5.84
SGK1	NM_005627		3.35	10.22	2.88	7.34	0.48	1.39
TFPI2	L27624		3.03	8.17	2.30	4.91	0.74	1.66
TENM1	AL022718		3.02	8.13	2.05	4.15	0.97	1.96
MSN	NM_002444		2.96	7.78	1.31	2.47	1.65	3.15
CDKN1A	NM_000389		2.83	7.11	1.84	3.58	0.99	1.99
VIP	NM_003381		2.82	7.08	2.55	5.87	0.27	1.21
ZDHHC2	AK001608		2.69	6.44	2.52	5.75	0.16	1.12
ACTA2	NM_001613		2.47	5.53	1.59	3.00	0.88	1.84
TFPI2	AL574096		2.42	5.37	1.80	3.48	0.63	1.54
ID1	D13889		2.40	5.28	0.78	1.72	1.62	3.07
SPATA18	AI559300		2.37	5.17	1.13	2.18	1.25	2.37
FRMD3	BF589413		2.24	4.73	1.68	3.21	0.56	1.47
FN1	AK026737		2.21	4.63	2.20	4.61	0.01	1.00
ZDHHC2	AI814257		2.21	4.63	2.00	3.99	0.21	1.16
FN1	BC005858		2.20	4.61	2.67	6.36	−0.46	1.38
SLC35F1	AI809083		2.17	4.51	1.46	2.74	0.72	1.64
KITLG	AI446414		2.16	4.48	1.55	2.94	0.61	1.53
IL13RA1	U81380		2.07	4.21	1.91	3.77	0.16	1.12
**DOWNREGULATED GENES**								
CARTPT	NM_004291		−3.36	10.25	−3.48	11.14	0.12	1.09
TMEM100	NM_018286		−3.15	8.90	−0.70	1.62	−2.46	5.48
AJAP1	AF052109		−2.84	7.17	−1.30	2.46	−1.54	2.92
ID4	AL022726		−2.77	6.81	−0.74	1.67	−2.03	4.09
HIF3A	BC026308		−2.51	5.71	−0.55	1.47	−1.96	3.89
SLC8A1	AI741439		−2.51	5.70	−0.64	1.56	−1.87	3.66
TXNIP	NM_006472		−2.45	5.48	−1.70	3.24	−0.76	1.69
IGF2	X07868		−2.45	5.46	−3.00	8.00	0.55	1.47
KIT	NM_000222		−2.41	5.32	−0.41	1.33	−2.00	4.00
LPAR1	AW269335		−2.34	5.07	−1.33	2.51	−1.01	2.02
TMEM178A	AA058832		−2.31	4.96	−0.43	1.34	−1.88	3.69
LINC01105	NM_153011		−2.25	4.77	−2.07	4.21	−0.18	1.13
WNT2B	BF961733		−2.25	4.75	−0.25	1.19	−1.99	3.98
SEMA6A	AB002438		−2.25	4.75	−2.11	4.31	−0.14	1.10
IGFBP7	NM_001553		−2.20	4.59	−2.64	6.23	0.44	1.36
GRIA2	NM_000826		−2.15	4.43	−1.22	2.33	−0.93	1.91
AJAP1	AA835004		−2.11	4.31	−0.29	1.23	−1.82	3.52
DMD	NM_004010		−2.10	4.29	−1.54	2.91	−0.56	1.47
NPY2R	U32500		−2.04	4.12	−1.50	2.83	−0.54	1.46

*Genes ordered for High expressing vs. Untransfected, compared to other comparison pairs*.

**Table 5:** Differential Genes expressed by FtMt overexpressing SH-SY5Y neuronal cells.

**GeneSymbol**	**RefSeq**		**Log2**	**Fold**	**Log2**	**Fold**	**Log2**	**Fold**
			**Diff**	**Change**	**Diff**	**Change**	**Diff**	**Change**
			***Med vs. Un***		***HighB vs. Un***		***HighB vs. Med***	
**UPREGULATED GENES**								
CALCA	BF447272		5.61	48.77	1.26	2.40	−4.34	20.31
CALCB	AA747379		3.83	14.22	0.58	1.49	−3.26	9.55
RGS13	AF030107		2.95	7.73	2.07	4.20	−0.88	1.84
SGK1	NM_005627		2.88	7.34	3.35	10.22	0.48	1.39
FN1	BC005858		2.67	6.36	2.20	4.61	−0.46	1.38
PLK2	NM_006622		2.59	6.02	1.71	3.27	−0.88	1.84
VIP	NM_003381		2.55	5.87	2.82	7.08	0.27	1.21
ZDHHC2	AK001608		2.52	5.75	2.69	6.44	0.16	1.12
RGS13	BC036950		2.50	5.64	1.78	3.44	−0.71	1.64
TMEM74	BC030710		2.48	5.58	−0.43	1.35	−2.91	7.53
CPED1	BF724137		2.47	5.55	1.64	3.12	−0.83	1.78
TFPI2	L27624		2.30	4.91	3.03	8.17	0.74	1.66
PRKCH	NM_006255		2.29	4.91	1.59	3.02	−0.70	1.62
EDIL3	AA053711		2.29	4.89	1.05	2.08	−1.23	2.35
FN1	AK026737		2.20	4.61	2.21	4.63	0.01	1.00
SMIM3	AF313413		2.14	4.40	0.96	1.94	−1.18	2.26
IGSF11	BE221674		2.12	4.34	1.21	2.31	−0.91	1.88
EGR1	AI459194		2.07	4.19	2.03	4.09	−0.03	1.02
TENM1	AL022718		2.05	4.15	3.02	8.13	0.97	1.96
**DOWNREGULATED GENES**								
CARTPT	NM_004291		−3.48	11.14	−3.36	10.25	−3.48	11.14
IGF2 /// INS–IGF2	X07868		−3.00	8.00	−2.45	5.46	−3.00	8.00
DLK1	U15979		−2.88	7.35	−2.63	6.19	−2.88	7.35
NAALAD2	BC038840		−2.74	6.69	−1.29	2.44	−2.74	6.69
IGFBP7	NM_001553		−2.64	6.23	−2.20	4.59	−2.64	6.23
SOX9	NM_000346		−2.33	5.03	−1.96	3.88	−2.33	5.03
INSM2	AA046951		−2.32	5.01	−1.54	2.90	−2.32	5.01
ABI3BP	AB056106		−2.25	4.77	−1.55	2.94	−2.25	4.77
KCNQ5	BF513800		−2.18	4.55	−1.32	2.50	−2.18	4.55
SEMA6A	AB002438		−2.11	4.31	−2.25	4.75	−2.11	4.31
SPOCK1	AF231124		−2.09	4.25	−2.00	4.01	−2.09	4.25
HIST1H4	NM_003542		−1.99	3.97	−0.62	1.54	−1.99	3.97
UTRN	N66570		−1.97	3.93	−1.00	2.00	−1.97	3.93
AQP1	AL518391		−1.96	3.88	−1.31	2.48	−1.96	3.88
PDZRN3	AL569804		−1.93	3.81	−0.85	1.81	−1.93	3.81
ZNF229	AA180985		−1.91	3.77	−0.75	1.68	−1.91	3.77
SLC16A10	AI935541		−1.86	3.64	−1.19	2.28	−1.86	3.64
SYTL4	AL391688		−1.82	3.54	−1.68	3.21	−1.82	3.54
SCAF11	AA679858		−1.81	3.50	−1.83	3.56	−1.81	3.50

*Genes ordered for Medium expressing vs. Untransfected, compared to other comparison pairs*.

**Table 6:** Differential Genes expressed by FtMT overexpressing SH-SY5Y neuronal cells.

**GeneSymbol**	**RefSeq**		**Log2**	**Fold**	**Log2**	**Fold**	**Log2**	**Fold**
			**Diff**	**Change**	**Diff**	**Change**	**Diff**	**Change**
			***HighB vs. Med***		***HighB vs. Un***		***Med vs. Un***	
**UPREGULATED GENES**								
GDF15	AF003934		2.55	5.84	4.39	21.02	1.85	3.60
FAM162B	AI540210		2.13	4.37	0.96	1.94	−1.17	2.25
TNFRSF10A	W65310		1.90	3.72	0.64	1.56	−1.25	2.38
NHLH2	AA166895		1.82	3.54	1.54	2.91	−0.28	1.22
MBNL3	AI197932		1.79	3.46	1.34	2.53	−0.46	1.37
CD99	NM_002414		1.70	3.24	0.51	1.43	−1.18	2.27
GALNT6	NM_007210		1.66	3.15	0.43	1.35	−1.23	2.34
MSN	NM_002444		1.65	3.15	2.96	7.78	1.31	2.47
SFXN4	AI346445		1.63	3.09	0.36	1.29	−1.26	2.40
ID1	D13889		1.62	3.07	2.40	5.28	0.78	1.72
RBPMS2	BE348466		1.55	2.93	0.96	1.95	−0.59	1.51
ITGB5	NM_002213		1.55	2.92	0.10	1.07	−1.44	2.72
CD99	U82164		1.54	2.92	0.50	1.41	−1.05	2.07
MYBL2	NM_002466		1.51	2.86	0.59	1.51	−0.92	1.89
ITPR1	L38019		1.49	2.81	1.22	2.33	−0.27	1.21
PTPRM	BC029442		1.48	2.78	0.28	1.21	−1.20	2.30
RIT2	NM_002930		1.46	2.76	0.74	1.67	−0.72	1.65
NAALAD2	BC038840		1.46	2.74	−1.29	2.44	−2.74	6.69
OSBPL3	AI202969		1.45	2.73	1.99	3.97	0.54	1.46
LYN	NM_002350		1.44	2.71	0.61	1.53	−0.82	1.77
**DOWNREGULATED GENES**								
CALCA	BF447272		−4.34	20.31	1.26	2.40	5.61	48.77
PPP2R2B	AA974416		−3.37	10.31	−1.58	2.98	1.79	3.46
CALCB	AA747379		−3.26	9.55	0.58	1.49	3.83	14.22
TMEM74	BC030710		−2.91	7.53	−0.43	1.35	2.48	5.58
NEGR1	AI123532		−2.65	6.26	−1.57	2.97	1.08	2.11
LDLRAD4	NM_004338		−2.51	5.69	−0.97	1.96	1.54	2.91
TMEM100	NM_018286		−2.46	5.48	−3.15	8.90	−0.70	1.62
PLD5	R38585		−2.24	4.74	−1.16	2.23	1.09	2.13
MMP28	NM_024302		−2.08	4.24	−0.58	1.49	1.50	2.84
ID4	AL022726		−2.03	4.09	−2.77	6.81	−0.74	1.67
KIT	NM_000222		−2.00	4.00	−2.41	5.32	−0.41	1.33
WNT2B	BF961733		−1.99	3.98	−2.25	4.75	−0.25	1.19
HIF3A	BC026308		−1.96	3.89	−2.51	5.71	−0.55	1.47
GRIA3	AW294729		−1.95	3.86	−1.27	2.41	0.68	1.60
FAM19A4	AA757457		−1.92	3.79	−1.32	2.50	0.60	1.52
DST	AL049215		−1.90	3.72	−1.62	3.07	0.28	1.21
TMEM178A	AA058832		−1.88	3.69	−2.31	4.96	−0.43	1.34
CADPS	BE467579		−1.88	3.67	−1.25	2.37	0.63	1.55
MRAP2	AA418816		−1.88	3.67	−1.06	2.08	0.82	1.76
SLC8A1	AI741439		−1.87	3.66	−2.51	5.70	−0.64	1.56

*Genes ordered for High B expressing vs. Medium, compared to other comparison pairs*.

#### Genes Associated With Neuronal Structure and Differentiation

Further data mining of the gene expression profiling data had revealed changes in a class of differentiation genes designated Inhibitor of Differentiation or Inhibitor of DNA-binding (ID) 1-4. These genes have been shown to be downregulated with RA treatment of neuroblastomas (Lopez-Carballo et al., 2002; Peddada et al., 2006). The data show increased expression between high expressor FtMt and untransfected neurons for ID1, ID2, and ID3, but reduced expression for ID4 (Table 7). For all of these listed genes, there were intermediate levels of expression in medium expressor FtMt clone. Table 7 also shows the gene expression results for a number of neuronal structural genes that were measured by western blot. Two tables of genes of interest related to iron metabolism and oxidative stress are also presented as Supplemental File 5). The majority of these genes did not show significant changes, but are involved in many of the key processes associated with FtMt function.

**Table 7:** Differential expression of genes associated with neuronal structure and differentiation.

**Gene Symbol**	**RefSeq**	**Intensity**	**Intensity**	**Intensity**	**Log2**	**Fold**
		***Un***	***Med***	***HighB***	***Un vs. HighB***	
ID1	D13889	168	289	889	1.7	5.3
ID2	NM_002166	786	1229	2038	0.95	2.6
ID3	NM_002167	181	551	1591	2.2	8.9
ID4	AW157094	520	409	172	−1.1	0.33
MYC	NM_002467	300	365	300	0	1
MYCN	BC002712	821	335	248	−1.2	0.3
PCNA	NM_002592	10,090	7,301	12,037	0.18	1.2
NEFL	AL566528	1,785	2,116	1,543	−0.14	0.86
**NEFM**	**NM_005382**	**11,227**	**16,853**	**16,335**	**0.38**	**1.45**
NEFH	NM_021076	1,076	905	1,249	0.37	1.45
**SNAP25**	**L19760**	**3,932**	**4.469**	**4,322**	**0.15**	**1.16**
**MAPT**	**J03778**	**1,117**	**1,763**	**1,053**	**−0.06**	**0.94**
MAP2	BF342661	7,218	6,353	7,384	0.02	1.02
TH	NM_000360	113	ND	ND	ND
DBH	NM_000787	5,988	2,991	6,630	0.1	1.1
**APP**	**NM_000484**	**4,606**	**4,285**	**3,415**	**−0.3**	**0.74**
**SNCA**	**NM_000345**	**468**	**981**	**569**	**0.2**	**1.2**

*Data shows raw intensity values (Intensity) between untransfected (Un), Medium FtMt expressing clone (Med) and High FtMt expressing clone (HighB)*.

*Log_2_ difference and Fold change between untransfected (Un) and High B clone are included to indicate which genes met the 1log_2_ difference criteria used for differential expression*.

*ND: Not detected*.

*Results in bold refer to genes that were analyzed at protein level by western blot*.

#### Validation of Genes Associated With Oxidative Stress

**Figure 7:**
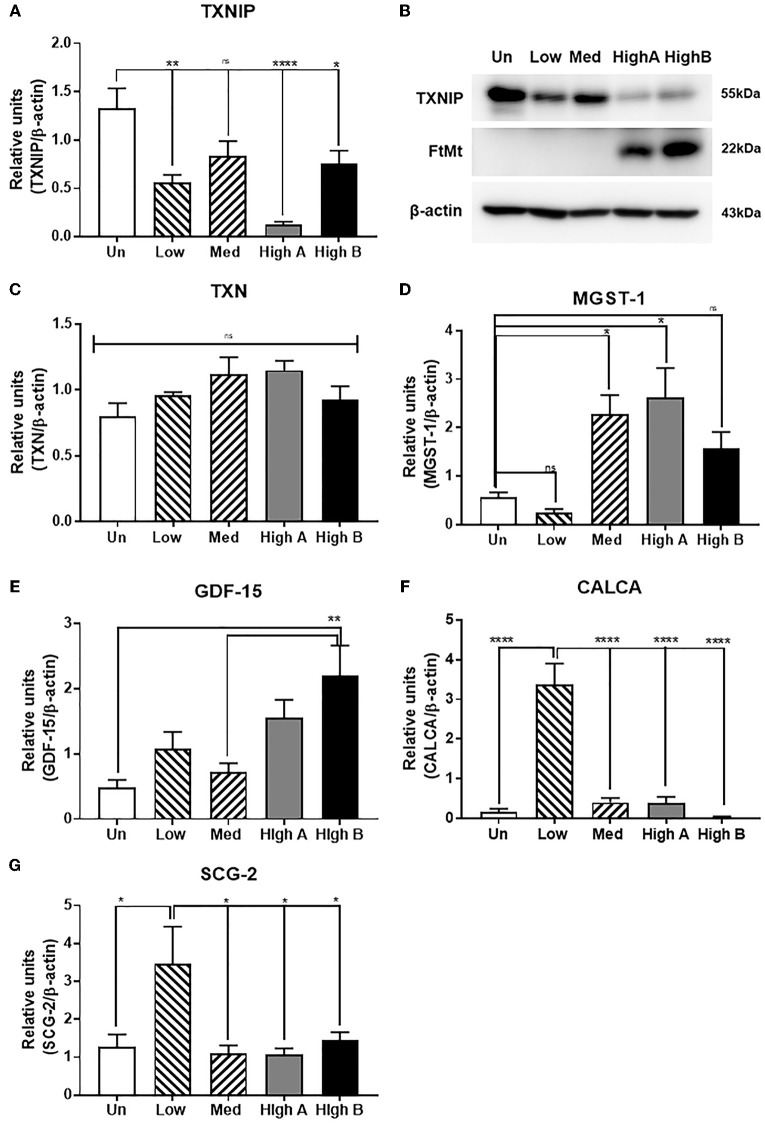
Validation of expression of key differentially expressed genes identified by microarray. **(A)** Real time PCR measurement of expression of thioredoxin-interacting protein (TXNIP) in different clones. Results are combined of three independent experiments. Data analyzed by one-way ANOVA with Tukey's *post-hoc* test of significance. Results indicate level of significance. *p < 0.05; **p < 0.01, ***p < 0.001, ****p < 0.0001. **(B)** Western blot showing relative levels of TXNIP protein in different clones compared to expression of FtMt and β-actin. ns: not significantly different **(C)** Real time PCR measurement of expression of thioredoxin (TXN) in different clones. Results are combined of three independent experiments. Data show no significant differences between expression levels of different clones. **(D)** Real time PCR measurement of expression of microsomal glutathione transferase (MGST)−1 in different clones. Results are combined of three independent experiments. Data analyzed by one-way ANOVA with Tukey's *post-hoc* test of significance. Results indicate level of significance. *p < 0.05; **p < 0.01. **(E)** Real time PCR measurement of expression of growth differentiation factor-15 (GDF-15) in different clones. Results are combined of three independent experiments. Data analyzed by one-way ANOVA with Tukey *post-hoc* test of significance. Results indicate level of significance. **p < 0.01. **(F)** Real time PCR measurement of expression of calcitonin gene related peptide alpha (CALCA) in different clones. Results are combined of three independent experiments. Data analyzed by one-way ANOVA with Tukey's *post-hoc* test of significance. Results indicate level of significance. **p < 0.01. **(G)** Real time PCR measurement of expression of secretogranin-2/chromogranin C (SCG-2) in different clones. Results are combined of three independent experiments. Data analyzed by one-way ANOVA with Tukey's *post-hoc* test of significance. Results indicate level of significance. *p < 0.05.

Although there are multiple criteria available for selecting genes of interest to study further, one of the central goals of the experiments was to investigate novel mechanisms associated with FtMt protection from oxidative stress. With this in mind, examination of the expression data indicated significant changes in expression of thioredoxin-interacting protein (TXNIP). This protein has multiple functions, but one key role is binding and inactivating reduced thioredoxin, a major cellular anti-oxidant. Increased amounts of cellular TXNIP will reduce the protective effects of thioredoxin. Gene expression data indicated relatively high expression in untransfected SH-SY5Y neurons, and considerable reduction in FtMt expressing clones. The highest reduction in TXNIP mRNA was in the high FtMt expressor, with less reduced in medium FtMt expressor, compared to untransfected cells. Three separate analysis for TXNIP were carried out on the microarray using separate probe sequences, but all showed similar results with reduction ranging from 5.5 fold to 3.8 fold. To confirm these findings, qPCR validation was carried out for TXNIP and TXN1 (Figures 7A,C). It is noticeable the considerable difference in TXNIP expression between the two different high FtMt expression clones. Both confirmed considerable reduction compared to untransfected cells (Figure 7A). For TXN, consistent with the microarray data that indicated a small non-significant increase in TXN expression in the high expressor clone, PCR validation produced similar result (Figures 7B,C); confirming that TXN expression is relatively unchanged. This is consistent with a protective mechanism involving reduced TXNIP levels. We have observed these differences in expression patterns between the two high expressor clones for TXNIP as well as some other genes that have to be further investigated. A western blot for TXNIP protein in these cells confirmed highest levels in untransfected cells, and least in the high expressor clones (High A and High B) (Figure 7B).

Another gene target of interest was microsomal glutathione transferase-1 (MGST1), which is localized to mitochondria, and is protective from oxidative stress (Figure 7D). Increased expression of MGST1 would also be protective from oxidative stress. Although the microarray data showed significant differences in expression of transferrin receptor (TFRC) between clones, this was not validated by qPCR analyses (data not shown).

#### Validation of Growth Factor Associated Genes

Three genes belonging to different classes of growth factor/neuroendocrine factors with the significant levels of differential expression (see Tables 4–6) were identified for validation. The genes GDF15 (growth differentiation factor 15), CALCA, SCG2, and CHGA share some common functions but were shown to have different expression profiles by microarray. Changes in expression of GDF15 (Figure 7E), CALCA (Figure 7F), and SCG2 (Figure 7G) for separate clones are shown with indicated significance between groups share common functions.

## Discussion

A number of studies have demonstrated that increased levels of FtMt in cells have significant protective effect from a range of insults linked to production of ROS, including those associated with neurodegenerative disease mechanisms. These previous studies have shown that protection can be conferred even with small levels of increased FtMt (Shi et al., 2010; Wang Y. Q. et al., 2016; You et al., 2016; Gao et al., 2017; Li J. et al., 2017). Although expressed at low levels compared to ubiquitious iron binding proteins such as ferritin, the protective effect would appear to come from its specific localization to mitochondria, the major cellular source of ROS (Nesi et al., 2017).

The significant protective effects of FtMt from insults associated with AD and PD have indicated that this protein might be a therapeutic target for these diseases. Gene delivery methods are feasible for targeted delivery to involved tissues, but it does raise the question about the consequences of overexpression of a protein that could be toxic to mitochondria. Several studies have utilized the widely used SH-SY5Y neuroblastoma cell as a model to study effects of FtMt, but all of these studies used undifferentiated cells. Some studies employed the mouse FtMt gene for overexpression in SH-SY5Y cells (Shi et al., 2010). The cleaved FtMt proteins only show 88% homology at the amino acid level and within this sequence, the human FtMt only has 2 cysteines while the mouse FtMt protein has 4. This difference can not only affect the redox properties of the protein but also its secondary structure. Because of these features, some differences in function are possible. For this study, we attempted to advance the previous models by establishing stable overexpression of human FtMt in SH-SY5Y, and then study them in a differentiated state as a simplified model of human neurons. Earlier studies have shown increase in mature neuronal features if SH-SY5Y cells were differentiated with retinoic acid (Nicolini et al., 1998; Simpson et al., 2001; Lopez-Carballo et al., 2002), and that differentiated SH-SY5Y neurons are more resistant to oxidative stress inducing toxins (Cecchi et al., 2008; Cheung et al., 2009; Lee et al., 2015; Forster et al., 2016) than undifferentiated cells. Retinoic acid differentiated SH-SY5Y cells were shown to express a significantly larger panel of mature neuronal genes (Pezzini et al., 2017).

In this study, we isolated a number of FtMt-expressing SH-SY5Y clones. Some of the clones proved unstable, but several clones that had relatively stable levels of FtMt expression after 2-3 passages showed normal cell growth and replicated at a significantly higher rate than untransfected cells. This finding is in contrast to one study that concluded that FtMt overexpression slowed the rate of neuroblastoma cell division (Shi et al., 2015). The overexpressing clones produced neurites at a lower density than untransfected cells. High FtMt expression did not affect the viability of cultures, but immunocytochemical staining for FtMt and the mitochondrial marker MtCox1 showed a higher number of aggregated mitochondria, a possible sign of mitochondrial damage. Analysis of nuclear, mitochondrial, and cytosolic fractions from the highest expressor FtMt clones showed that the majority of overexpressed FtMt was localized to mitochondria, though some could be detected in the nuclear fraction.

The value of the clones isolated for future studies is their expression of different levels of FtMt mRNA. The difference in expression ranged was 20-fold from lowest to highest. The lowest FtMt expressor expressed readily detectable mRNA by qPCR, but we were unable to detect protein in these cells. This is unexpected as the difference in mRNA expression levels would indicate that protein should be detectable. It is possible that some mechanism is involved in the rapid degradation of the low level of protein. This is being investigated, but to date the mechanisms of FtMt processing and degradation have not been identified. This hypothesis is strengthened if you consider the medium expressor clone, which expressed 3.9-fold less RNA than the highest expressor, but 22.1-fold less protein. Future experiments will include the use of inhibitors of proteosomal degradation and autophagy to determine if these affect cellular FtMt levels. Unlike in some other studies, the untransfected isolate of SH-SY5Y cells used to produce the transfected clones expressed no detectable FtMt RNA or protein. These experiments used a different SH-SY5Y isolate compared to experiments which we previously reported (Guan et al., 2017).

The characterization of FtMt overexpressing cells showed differences in expression of mature neuronal proteins. The differences in levels of NF-M, PSD-95, and SNAP-25, along with the disease-associated proteins APP, tau, and α-synuclein suggest changes in neuronal properties. Further studies will determine if FtMt overexpression affects cellular production of the Aβ peptide from APP, or the levels of phosphorylated tau or α-synuclein, which are associated with AD or PD pathology. The use of these experimental models of stable FtMt overexpression for studying changes in aggregation or phosphorylation of tau and α-synuclein would be feasible.

A potentially significant new finding from the gene array data has been linking overexpression of FtMt to downregulation of TXNIP expression. TXNIP is a member of the alpha arrestin protein family that regulates and binds to reduced thioredoxin (TXN) and can be induced in response to oxidative stress, calcium influx and hyperglycemia (Kim et al., 2012). TXNIP has many identified functions, but is an important regulator of thioredoxin activity. Thioredoxin, a thiol-oxidoreductase, is a major regulator of cellular redox signaling which protects cells from oxidative stress (Mahmood et al., 2013). Binding of thioredoxin and TXNIP inhibits the anti-oxidative function resulting in the greater accumulation of reactive oxygen species and cellular stress. Increased levels of TXNIP has been associated with many diseases of oxidative stress including ischemic heart disease, diabetes, cancer, AD, and PD (Zhou et al., 2010; Li J. et al., 2017; Duan et al., 2018; Melone et al., 2018). Thioredoxin-TXNIP is a major regulator of mitochondrial-mediated oxidative stress in many pathologies including cerebrovascular and neurodegenerative diseases (Nasoohi et al., 2018). Areas with the highest level of thioredoxin activity in brain are those with the highest level of metabolic and oxidative burden (Aon-Bertolino et al., 2011), similar to FtMt. One recent study that linked TXNIP to AD pathology showed that inhibiting TXNIP expression in SH-SY5Y cells and AD model mice using a pharmaceutical agent directly inhibited the phosphorylation of the microtubule associated protein tau (Melone et al., 2018). Increased phosphorylation of tau is a key pathological event in AD, and its inhibition due to reduced TXNIP expression that can be caused by FtMt overexpression could provide further rationale forthis therapeutic approach for AD.

TXNIP expression is induced in neurons after oxidative or glucose stress in either ischemic or hyperglycemic-ischemic condition (Kim et al., 2012). Induction of TXNIP can be pro-apoptotic under these conditions. The changes in TXNIP protein levels in these overexpressing clones followed the same pattern as TXNIP mRNA expression. Downregulation of TXNIP while maintaining levels of thioredoxin-1 and –2 will provide protection from oxidative stress. Another factor shown to be protective is microsomal glutathione transferase-1 (MGST1), which accumulates in mitochondria and is protective from oxidative stress (Siritantikorn et al., 2007). It has been observed that MGST1 expression was very low in neuroblastomas and neuroblastoma cell line (Bjorkhem-Bergman et al., 2014). These authors hypothesized that this could explain the extreme sensitivity of neuroblastomas to oxidative stress. Increasing MGST1 expression resulted in significant protection of MCF-2 cells from lipid peroxidation (Siritantikorn et al., 2007), while downregulating expression increased PC12 cells susceptibility to toxic stress (Sobczak et al., 2014).

Three other groups of genes that coded for growth factor and cytokine molecules were investigated as they had shown significant differences between FtMt expressing clones by microarray analysis. Growth differentiation factor-15 (GDF-15), also called macrophage inhibitory cytokine-1 (MIC-1), was strongly upregulated in FtMt expressing clones. It was initially identified as a powerful protective factor for cultured dopaminergic neurons exposed to toxic doses of iron and other toxins (Strelau et al., 2000, 2003). Deficiency of GDF-15 has been shown to increase the vulnerability of dopaminergic neurons to 6-OHDA administration in mice (Machado et al., 2016). GDF-15 was shown to have a role in normal erythropoiesis, and its expression was increased in erythroblasts from patients with refractory anemia, which is associated with large increase in iron in mitochondria (Ramirez et al., 2009). GDF-15 can regulate energy homeostasis in mitochondria in muscle through elevation of oxidative metabolism (Cheung et al., 2009). Expression of calcitonin gene related polypeptide alpha (CALCA) and beta (CALCB) genes were significantly altered. These genes produce pro-peptides that are cleaved to neuroactive peptide hormones calcitonin and calcitonin gene related polypeptide (CGRP), which are involved in calcium homeostasis, including vasodilation. CGRP expression is high in the CNS suggesting neurotransmitter activity. CGRP was shown to provide significant protective properties in different experimental systems, including heat injury and ischemia/reperfusion in rats and overexpression in Schwann cells subject to oxidative stress (Russell et al., 2014; Wu et al., 2015; Yang et al., 2016; Lu et al., 2017). Secretogranin II (SCG2)(Chromagranin C) is also a neuroendrocrine secretory protein that is cleaved to form the peptide secretoneurin. Expression of SCG2 is modulated by extracellular calcium (Zhan et al., 2008), and it can induce expression of the anti-apoptotic protein Bcl2 through activation of JAK2/STAT3 signaling, providing protection in a murine stroke model (Shyu et al., 2008). This gene is involved in neuronal differentiation and resistance to apoptosis. Due to the large amount of data generated by the microarray profiling of different FtMt clones, there are a number of other genes of potential interest to neuronal phenotype and differentiation that can be explored.

In conclusion, our data have identified that FtMt overexpression has significant effects on neuronal-like cells. The results indicate that the protective effect is not dependent on amount of FtMt expression. High levels of expression did not appear to harm the viability of cells in terms of growth and differentiation, but some mitochondria abnormalities were observed in the highest expressing clone. There was a clear difference in expression of many genes between low and high expressing clones that suggest a dose effect. cells. The data provides a framework for investigating novel mechanisms concerning FtMt expression in diseases such as AD, and whether FtMt could be considered as a candidate for use in gene therapy for diseases involving mitochondrial oxidative stress.

## Author Contributions

AM performed all experiments, analyzed data and assisted in manuscript preparation. ST assisted in experiments and data acquisition. DGW planned experiments, supervised experiments, assisted in data analyses and wrote manuscript. IT initiated experiments, provided funding and intellectual input to data interpretation and manuscript. All authors read and approved all versions of manuscript.

### Conflict of Interest Statement

The authors declare that the research was conducted in the absence of any commercial or financial relationships that could be construed as a potential conflict of interest.

## Supplementary Material

Supplemental Figure 1 Western blot panel showing FtMt expression in all isolated clones of SH-SY5Y cells transfected with FtMt expression plasmid. Results are shown for FtMt expression in undifferentiated cells.

Supplemental File 1 Zipped Microsoft Excel File showing complete results of all expression intensities for analyzed clones. File is searchable.

Supplemental File 2 Microsoft Excel File complete results of expression intensities with changes up or down by more than 1 log2 comparing between highest expressing clone (HighB) and untransfected cells.

Supplemental File 3 Microsoft Excel File complete results of expression intensities with changes up or down by more than 1 log2 comparing between medium expressing clone and untransfected cells.

Supplemental File 4 Microsoft Excel File complete results of expression intensities with changes up or down by more than 1 log2 comparing between highest expressing (high B) and medium FtMt expressing clones.

Supplemental File 5 Microsoft word table listing expression differences of key genes related to Iron Metabolism and to Oxidative Stress.

## References

[B1] Aon-Bertolino M. L. Romero J. I. Galeano P. Holubiec M. Badorrey M. S. Saraceno G. E.. (2011). Thioredoxin and glutaredoxin system proteins-immunolocalization in the rat central nervous system. Biochim. Biophys. Acta 1810, 93–110. 10.1016/j.bbagen.2010.06.011 20620191

[B2] Arosio P. Ingrassia R. Cavadini P. (2009). Ferritins: a family of molecules for iron storage, antioxidation and more. Biochim. Biophys. Acta 1790, 589–599. 10.1016/j.bbagen.2008.09.004 18929623

[B3] Bjorkhem-Bergman L. Johansson M. Morgenstern R. Rane A. Ekstrom L. (2014). Prenatal expression of thioredoxin reductase 1 (TRXR1) and microsomal glutathione transferase 1 (MGST1) in humans. FEBS Open Bio 4, 886–891. 10.1016/j.fob.2014.10.005 25379386 PMC4215115

[B4] Brundin P. Dave K. D. Kordower J. H. (2017). Therapeutic approaches to target alpha-synuclein pathology. Exp. Neurol. 298, 225–235. 10.1016/j.expneurol.2017.10.003 28987463 PMC6541231

[B5] Bustin S. A. Benes V. Garson J. A. Hellemans J. Huggett J. Kubista M.. (2009). The MIQE guidelines: minimum information for publication of quantitative real-time PCR experiments. Clin. Chem. 55, 611–622. 10.1373/clinchem.2008.112797 19246619

[B6] Cecchi C. Pensalfini A. Liguri G. Baglioni S. Fiorillo C. Guadagna S.. (2008). Differentiation increases the resistance of neuronal cells to amyloid toxicity. Neurochem. Res. 33, 2516–2531. 10.1007/s11064-008-9627-7 18307032

[B7] Cheung Y.-T. Lau W. K.-W. Yu M.-S. Lai C. S.-W. Yeung S.-C. So K.-F.. (2009). Effects of all-trans-retinoic acid on human SH-SY5Y neuroblastoma as in vitro model in neurotoxicity research. Neurotoxicology 30, 127–135. 10.1016/j.neuro.2008.11.001 19056420

[B8] Drysdale J. Arosio P. Invernizzi R. Cazzola M. Volz A. Corsi B. (2002). Mitochondrial ferritin: a new player in iron metabolism. *Blood Cells. Mol*. Dis. 29, 376–383. 10.1006/bcmd.2002.0577 12547228

[B9] Duan J. Du C. Shi Y. Liu D. Ma J. (2018). Thioredoxin-interacting protein deficiency ameliorates diabetic retinal angiogenesis. Int. J. Biochem. Cell Biol. 94, 61–70. 10.1016/j.biocel.2017.11.013 29203232

[B10] Forster J. I. Koglsberger S. Trefois C. Boyd O. Baumuratov A. S. Buck L. (2016). Characterization of differentiated SH-SY5Y as neuronal screening model reveals increased oxidative vulnerability. J. Biomol. Screen. 21, 496–509. 10.1177/1087057115625190 26738520 PMC4904349

[B11] Gao G. Chang Y.-Z. (2014). Mitochondrial ferritin in the regulation of brain iron homeostasis and neurodegenerative diseases. Front. Pharmacol. 5:19. 10.3389/fphar.2014.00019 24596558 PMC3925988

[B12] Gao G. Zhang N. Wang Y.-Q. Wu Q. Yu P. Shi Z.-H.. (2017). Mitochondrial ferritin protects hydrogen peroxide-induced neuronal cell damage. Aging Dis. 8, 458–470. 10.14336/AD.2016.1108 28840060 PMC5524808

[B13] Gong S. Chen Y. Meng F. Zhang Y. Wu H. Wu F. (2017). Roflumilast restores cAMP/PKA/CREB signaling axis for FtMt-mediated tumor inhibition of ovarian cancer. Oncotarget 8, 112341–112353. 10.18632/oncotarget.22866 29348829 PMC5762514

[B14] Guan H. Yang H. Yang M. Yanagisawa D. Bellier J.-P. Mori M.. (2017). Mitochondrial ferritin protects SH-SY5Y cells against H2O2-induced oxidative stress and modulates alpha-synuclein expression. Exp. Neurol. 291, 51–61. 10.1016/j.expneurol.2017.02.001 28163159

[B15] Guaraldo M. Santambrogio P. Rovelli E. Di Savino A. Saglio G. Cittaro D.. (2016). Characterization of human mitochondrial ferritin promoter: identification of transcription factors and evidences of epigenetic control. Sci. Rep. 6:33432. 10.1038/srep33432 27625068 PMC5022048

[B16] Huang M. L.-H. Becker E. M. Whitnall M. Suryo Rahmanto Y. Ponka P. Richardson D. R. (2009). Elucidation of the mechanism of mitochondrial iron loading in Friedreich's ataxia by analysis of a mouse mutant. Proc. Natl. Acad. Sci. U.S.A. 106, 16381–16386. 10.1073/pnas.0906784106 19805308 PMC2752539

[B17] Jan A. T. Azam M. Rahman S. Almigeiti A. M. S. Choi D. H. Lee E. J.. (2017). Perspective insights into disease progression, diagnostics, and therapeutic approaches in alzheimer's disease: a judicious update. Front. Aging Neurosci. 9:356. 10.3389/fnagi.2017.00356 29163138 PMC5671974

[B18] Kim G. S. Jung J. E. Narasimhan P. Sakata H. Chan P. H. (2012). Induction of thioredoxin-interacting protein is mediated by oxidative stress, calcium, and glucose after brain injury in mice. Neurobiol. Dis. 46, 440–449. 10.1016/j.nbd.2012.02.008 22366181 PMC3323710

[B19] Lang A. E. Espay A. J. (2018). Disease modification in Parkinson's disease: current approaches, challenges, and future considerations. Mov. Disord. 33, 660–677. 10.1002/mds.27360 29644751

[B20] Lee C.-I. Perng J.-H. Chen H.-Y. Hong Y.-R. Wang J.-J. (2015). Undifferentiated neuroblastoma cells are more sensitive to photogenerated oxidative stress than differentiated cells. J. Cell. Biochem. 116, 2074–2085. 10.1002/jcb.25165 25919890

[B21] Levi S. Arosio P. (2004). Mitochondrial ferritin. Int. J. Biochem. Cell Biol. 36, 1887–1889. 10.1016/j.biocel.2003.10.020 15203103

[B22] Levi S. Corsi B. Bosisio M. Invernizzi R. Volz A. Sanford D.. (2001). A human mitochondrial ferritin encoded by an intronless gene. J. Biol. Chem. 276, 24437–24440. 10.1074/jbc.C100141200 11323407

[B23] Li J. Yue Z. Xiong W. Sun P. You K. Wang J. (2017). TXNIP overexpression suppresses proliferation and induces apoptosis in SMMC7221 cells through ROS generation and MAPK pathway activation. Oncol. Rep. 37, 3369–3376. 10.3892/or.2017.5577 28440491

[B24] Li X. Wang P. Wu Q. Xie L. Cui Y. Li H.. (2017). The construction and characterization of mitochondrial ferritin overexpressing mice. Int. J. Mol. Sci. 18:1518. 10.3390/ijms18071518 28703745 PMC5536008

[B25] Lopez-Carballo G. Moreno L. Masia S. Perez P. Barettino D. (2002). Activation of the phosphatidylinositol 3-kinase/Akt signaling pathway by retinoic acid is required for neural differentiation of SH-SY5Y human neuroblastoma cells. J. Biol. Chem. 277, 25297–25304. 10.1074/jbc.M201869200 12000752

[B26] Lu C.-X. Qiu T. Liu Z.-F. Su L. Cheng B. (2017). Calcitonin gene-related peptide has protective effect on brain injury induced by heat stroke in rats. Exp. Ther. Med. 14, 4935–4941. 10.3892/etm.2017.5126 29201197 PMC5704302

[B27] Machado V. Haas S. J.-P. von Bohlen Und Halbach O. Wree A. Krieglstein K. Unsicker K.. (2016). Growth/differentiation factor-15 deficiency compromises dopaminergic neuron survival and microglial response in the 6-hydroxydopamine mouse model of Parkinson's disease. Neurobiol. Dis. 88, 1–15. 10.1016/j.nbd.2015.12.016 26733415

[B28] Mahmood D. F. D. Abderrazak A. El Hadri K. Simmet T. Rouis M. (2013). The thioredoxin system as a therapeutic target in human health and disease. Antioxid. Redox Signal. 19, 1266–1303. 10.1089/ars.2012.4757 23244617

[B29] Melone M. A. B. Dato C. Paladino S. Coppola C. Trebini C. Giordana M. T.. (2018). Verapamil Inhibits Ser202/Thr205 phosphorylation of tau by blocking TXNIP/ROS/p38 MAPK pathway. Pharm. Res. 35:44. 10.1007/s11095-017-2276-2 29404777

[B30] Nasoohi S. Ismael S. Ishrat T. (2018). Thioredoxin-Interacting Protein (TXNIP) in cerebrovascular and neurodegenerative diseases: regulation and implication. Mol. Neurobiol. 55, 7900–7920. 10.1007/s12035-018-0917-z 29488135 PMC6388721

[B31] Nesi G. Sestito S. Digiacomo M. Rapposelli S. (2017). Oxidative stress, mitochondrial abnormalities and proteins deposition: multitarget approaches in Alzheimer's disease. Curr. Top. Med. Chem. 17, 3062–3079. 10.2174/1568026617666170607114232 28595557

[B32] Nicolini G. Miloso M. Zoia C. Di Silvestro A. Cavaletti G. Tredici G. (1998). Retinoic acid differentiated SH-SY5Y human neuroblastoma cells: an *in vitro* model to assess drug neurotoxicity. Anticancer Res. 18, 2477–2481. 9703895

[B33] Peddada S. Yasui D. H. LaSalle J. M. (2006). Inhibitors of differentiation (ID1, ID2, ID3 and ID4) genes are neuronal targets of MeCP2 that are elevated in Rett syndrome. Hum. Mol. Genet. 15, 2003–2014. 10.1093/hmg/ddl124 16682435 PMC1931415

[B34] Pezzini F. Bettinetti L. Di Leva F. Bianchi M. Zoratti E. Carrozzo R.. (2017). Transcriptomic profiling discloses molecular and cellular events related to neuronal differentiation in SH-SY5Y neuroblastoma cells. Cell. Mol. Neurobiol. 37, 665–682. 10.1007/s10571-016-0403-y 27422411 PMC11482124

[B35] Ramirez J.-M. Schaad O. Durual S. Cossali D. Docquier M. Beris P.. (2009). Growth differentiation factor 15 production is necessary for normal erythroid differentiation and is increased in refractory anaemia with ring-sideroblasts. Br. J. Haematol. 144, 251–262. 10.1111/j.1365-2141.2008.07441.x 19036111

[B36] Ross R. A. Spengler B. A. Biedler J. L. (1983). Coordinate morphological and biochemical interconversion of human neuroblastoma cells. J. Natl. Cancer Inst. 71, 741–747. 6137586

[B37] Russell F. A. King R. Smillie S.-J. Kodji X. Brain S. D. (2014). Calcitonin gene-related peptide: physiology and pathophysiology. Physiol. Rev. 94, 1099–1142. 10.1152/physrev.00034.2013 25287861 PMC4187032

[B38] Santambrogio P. Biasiotto G. Sanvito F. Olivieri S. Arosio P. Levi S. (2007). Mitochondrial ferritin expression in adult mouse tissues. J. Histochem. Cytochem. 55, 1129–1137. 10.1369/jhc.7A7273.2007 17625226 PMC3957534

[B39] Shi Z.-H. Nie G. Duan X.-L. Rouault T. Wu W.-S. Ning B. Zhang N. Chang Y.-Z. Zhao B.-L (2010). Neuroprotective mechanism of mitochondrial ferritin on 6-hydroxydopamine-induced dopaminergic cell damage: implication for neuroprotection in Parkinson's disease. Antioxid. Redox Signal. 13, 783–796. 10.1089/ars.2009.3018 20121342 PMC6777976

[B40] Shi Z.-H. Shi F.-F. Wang Y.-Q. Sheftel A. D. Nie G. Zhao Y.-S.. (2015). Mitochondrial ferritin, a new target for inhibiting neuronal tumor cell proliferation. Cell. Mol. Life Sci. 72, 983–997. 10.1007/s00018-014-1730-0 25213357 PMC4323545

[B41] Shyu W.-C. Lin S.-Z. Chiang M.-F. Chen D.-C. Su C.-Y. Wang H.-J.. (2008). Secretoneurin promotes neuroprotection and neuronal plasticity via the Jak2/Stat3 pathway in murine models of stroke. J. Clin. Invest. 118, 133–148. 10.1172/JCI32723 18079966 PMC2129236

[B42] Simpson P. B. Bacha J. I. Palfreyman E. L. Woollacott A. J. McKernan R. M. Kerby J. (2001). Retinoic acid evoked-differentiation of neuroblastoma cells predominates over growth factor stimulation: an automated image capture and quantitation approach to neuritogenesis. Anal. Biochem. 298, 163–169. 10.1006/abio.2001.5346 11757502

[B43] Siritantikorn A. Johansson K. Ahlen K. Rinaldi R. Suthiphongchai T. Wilairat P.. (2007). Protection of cells from oxidative stress by microsomal glutathione transferase 1. Biochem. Biophys. Res. Commun. 355, 592–596. 10.1016/j.bbrc.2007.02.018 17306223

[B44] Snyder A. M. Wang X. Patton S. M. Arosio P. Levi S. Earley C. J.. (2009). Mitochondrial ferritin in the substantia nigra in restless legs syndrome. J. Neuropathol. Exp. Neurol. 68, 1193–1199. 10.1097/NEN.0b013e3181bdc44f 19816198 PMC3024883

[B45] Sobczak M. Boczek T. Kowalski A. Wiktorska M. Niewiarowska J. Zylinska L. (2014). Downregulation of microsomal glutathione-S-transferase 1 modulates protective mechanisms in differentiated PC12 cells. J. Physiol. Biochem. 70, 375–383. 10.1007/s13105-014-0312-9 24419913

[B46] Stenirri S. Santambrogio P. Setaccioli M. Erba B. G. Pia Manitto M. Rovida E.. (2012). Study of FTMT and ABCA4 genes in a patient affected by age-related macular degeneration: identification and analysis of new mutations. Clin. Chem. Lab. Med. 50, 1021–1029. 10.1515/cclm-2011-0854 22706241

[B47] Strelau J. Bottner M. Lingor P. Suter-Crazzolara C. Galter D. Jaszai J. (2000). GDF-15/MIC-1 a novel member of the TGF-beta superfamily. J. Neural Transm. 60, 273–276. 10.1007/978-3-7091-6301-6_18 11205146

[B48] Strelau J. Schober A. Sullivan A. Schilling L. Unsicker K. (2003). Growth/differentiation factor-15 (GDF-15), a novel member of the TGF-beta superfamily, promotes survival of lesioned mesencephalic dopaminergic neurons *in vitro* and *in vivo* and is induced in neurons following cortical lesioning. J. Neural Transm. 65, 197–203. 10.1007/978-3-7091-0643-3_12 12946057

[B49] Walker D. G. Dalsing-Hernandez J. E. Campbell N. A. Lue L.-F. (2009). Decreased expression of CD200 and CD200 receptor in Alzheimer's disease: a potential mechanism leading to chronic inflammation. Exp. Neurol. 215, 5–19. 10.1016/j.expneurol.2008.09.003 18938162 PMC2765462

[B50] Wang L. Yang H. Zhao S. Sato H. Konishi Y. Beach T. G.. (2011). Expression and localization of mitochondrial ferritin mRNA in Alzheimer's disease cerebral cortex. PLoS ONE 6:e22325. 10.1371/journal.pone.0022325 21799823 PMC3140525

[B51] Wang P. Wu Q. Wu W. Li H. Guo Y. Yu P.. (2017). Mitochondrial ferritin deletion exacerbates beta-amyloid-induced neurotoxicity in mice. Oxid. Med. Cell. Longev. 2017:1020357. 10.1155/2017/1020357 28191272 PMC5278219

[B52] Wang X. Yang H. Yanagisawa D. Bellier J.-P. Morino K. Zhao S.. (2016). Mitochondrial ferritin affects mitochondria by stabilizing HIF-1alpha in retinal pigment epithelium: implications for the pathophysiology of age-related macular degeneration. Neurobiol. Aging 47, 168–179. 10.1016/j.neurobiolaging.2016.07.025 27599360

[B53] Wang Y.-Q. Chang S.-Y. Wu Q. Gou Y.-J. Jia L. Cui Y.-M.. (2016). The protective role of mitochondrial ferritin on erastin-induced ferroptosis. Front. Aging Neurosci. 8:308. 10.3389/fnagi.2016.00308 28066232 PMC5167726

[B54] Wu W.-S. Zhao Y.-S. Shi Z.-H. Chang S.-Y. Nie G.-J. Duan X.-L.. (2013). Mitochondrial ferritin attenuates beta-amyloid-induced neurotoxicity: reduction in oxidative damage through the Erk/P38 mitogen-activated protein kinase pathways. Antioxid. Redox Signal. 18, 158–169. 10.1089/ars.2011.4285 22746342

[B55] Wu Y. Hao G.-M. He J. Lv T.-T. Wang H.-L. Mao Y.-Q.. (2015). Lentivirus mediated over expression of CGRP inhibited oxidative stress in Schwann cell line. Neurosci. Lett. 598, 52–58. 10.1016/j.neulet.2015.05.009 25960317

[B56] Yang H. Guan H. Yang M. Liu Z. Takeuchi S. Yanagisawa D.. (2015). Upregulation of mitochondrial ferritin by proinflammatory cytokines: implications for a role in Alzheimer's disease. J. Alzheimers. Dis. 45, 797–811. 10.3233/JAD-142595 25624418

[B57] Yang H. Yang M. Guan H. Liu Z. Zhao S. Takeuchi S.. (2013). Mitochondrial ferritin in neurodegenerative diseases. Neurosci. Res. 77, 1–7. 10.1016/j.neures.2013.07.005 23916831

[B58] Yang M. Yang H. Guan H. Kato T. Mukaisho K. Sugihara H.. (2017). Characterization of a novel monoclonal antibody against human mitochondrial ferritin and its immunohistochemical application in human and monkey Substantia Nigra. Acta Histochem. Cytochem. 50, 49–55. 10.1267/ahc.16034 28386150 PMC5374103

[B59] Yang S. I. Yuan Y. Jiao S. Luo Q. I. Yu J. (2016). Calcitonin gene-related peptide protects rats from cerebral ischemia/reperfusion injury via a mechanism of action in the MAPK pathway. Biomed. Rep. 4, 699–703. 10.3892/br.2016.658 27284409 PMC4887836

[B60] You L.-H. Li Z. Duan X.-L. Zhao B.-L. Chang Y.-Z. Shi Z.-H. (2016). Mitochondrial ferritin suppresses MPTP-induced cell damage by regulating iron metabolism and attenuating oxidative stress. Brain Res. 1642, 33–42. 10.1016/j.brainres.2016.03.023 27017962

[B61] Yu X. Gao D. (2013). Overexpression of cytoglobin gene inhibits hypoxic injury to SH-SY5Y neuroblastoma cells. Neural Regen. Res. 8, 2198–2203. 10.3969/j.issn.1673-5374.2013.23.010 25206529 PMC4146124

[B62] Zhan X. Li F. Chu Q. Pang H. Kim H. J. Denli A. M. (2008). Cellular expression and subcellular localization of secretogranin II in the mouse hippocampus and cerebellum. Exp. Gerontol. 497, 53–63. 10.1016/j.bbrc.2018.02.130

[B63] Zhou R. Tardivel A. Thorens B. Choi I. Tschopp J. (2010). Thioredoxin-interacting protein links oxidative stress to inflammasome activation. Nat. Immunol. 11, 136–140. 10.1038/ni.1831 20023662

