# Supplementary figures and images for: Differences in Gene Expression Profiles and Phenotypes of Differentiated SH-SY5Y Neurons Stably Overexpressing Mitochondrial Ferritin
Source: Front Mol Neurosci. 2019 Jan 8;11:470. doi: 10.3389/fnmol.2018.00470 (PMC6331485; doi:10.3389/fnmol.2018.00470)

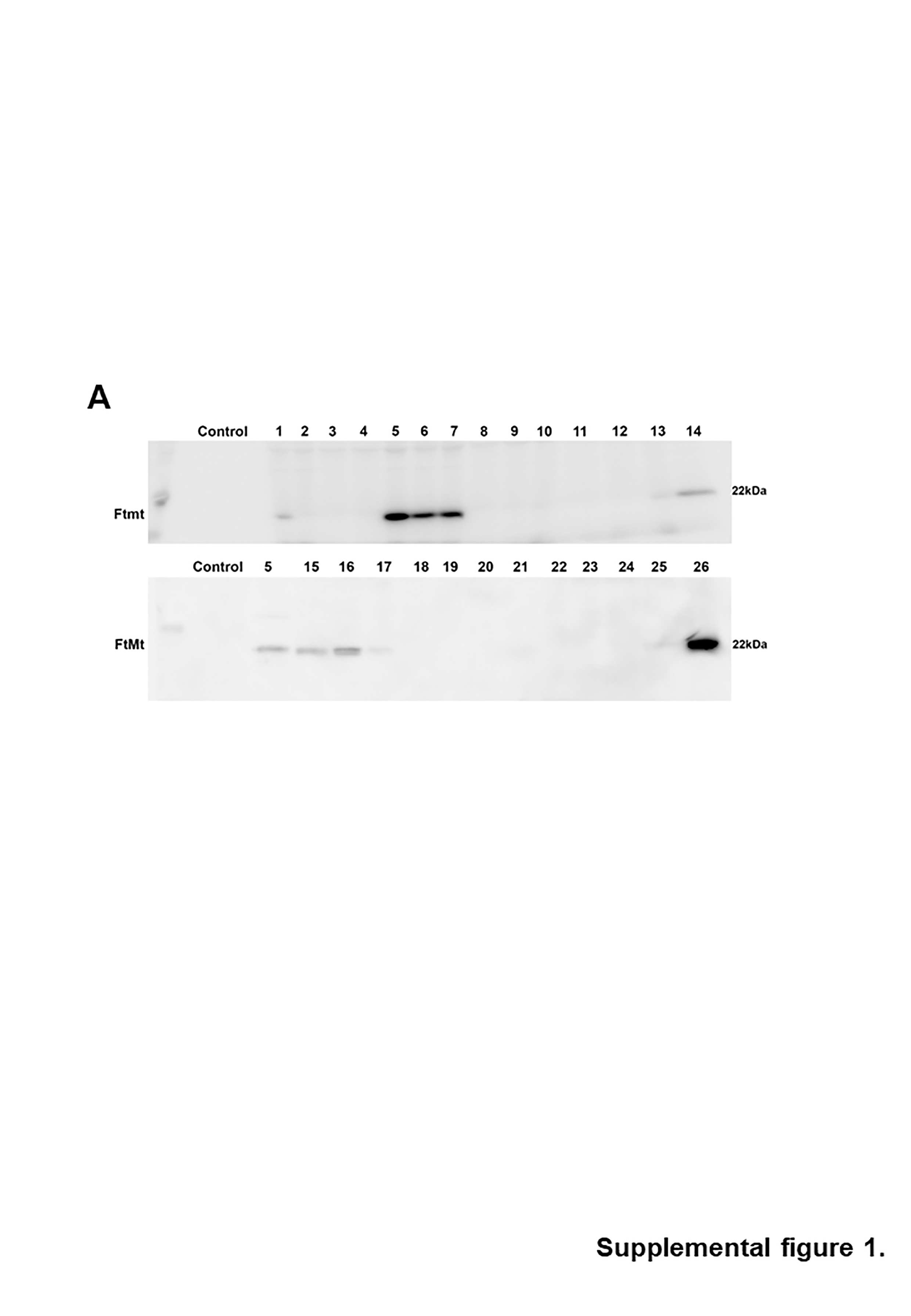

Supplement: Supplemental Figure 1 — Western blot panel showing FtMt expression in all isolated clones of SH-SY5Y cells transfected with FtMt expression plasmid. Results are shown for FtMt expression in undifferentiated cells. [file Image_1.tif]
