# Supplementary material for: Differences in Gene Expression Profiles and Phenotypes of Differentiated SH-SY5Y Neurons Stably Overexpressing Mitochondrial Ferritin
Source: Front Mol Neurosci. 2019 Jan 8;11:470. doi: 10.3389/fnmol.2018.00470 (PMC6331485; doi:10.3389/fnmol.2018.00470)
Supplement: Supplemental File 5 — Microsoft word table listing expression differences of key genes related to Iron Metabolism and to Oxidative Stress. [file Data_Sheet_5.docx]

**Supplemental File 5. Expression Differences of key Genes grouped according to function**

**Iron Metabolism**

**Gene Symbol RefSeq Intensity Intensity Intensity Log2 Fold**

*Un Med HighB Un vs HighB*

TF NM_001063 ND ND ND ND

TFRC NM_003234 5,389 7,026 11,093 1.04 2.06

TFR2 NM_003227 224 169 218 -0.04 1.03

STEAP3 NM_018234 137.3 73.8 148.7 0.12 1.08

SLC11A2 NM_000617 905 950 899 -0.01 1.01

FLVCR2 AW001026 143.6 ND ND ND

HMOX1 NM_002133 ND ND 123 ND

HMOX2 NM_002134 ND ND ND ND

LCN2 NM_005564 ND ND ND ND

FTH1 NM_002032 12,356 14,939 15,174 0.30 1.23

FTL BG538564 13,988 15,640 17,982 0.36 1.29

Fech NM_000140 1,029 1,119 1,277 0.31 1.24

ABCB10 AF277184 1,198 1,284 1,209 0.01 1.01

ABCB7 AB005289 893 756 903 0.02 1.01

ABCB6 NM_005689 818 781 740 -0.15 1.11

SLC25A37 NM_018579 414 388 362 -0.19 1.14

BDH2 NM_020139 1,761 1,618 1,506 -0.23 1.17

SLC40A1 AL136944 570 637 820 0.52 1.44

ACO1 NM_002197 754 759 769 0.03 1.02

IREB2 BF438417 4,278 4,130 3,561 -0.26 1.20

**Oxidative Stress**

**Gene Symbol RefSeq Intensity Intensity Intensity Log2 Fold**

*Un Med HighB Un vs HighB*

**TXNIP NM_006472 1,545 2,610 8,462 -2.45 5.48**

PDIA2 NM_006849 88 283 356 -2.01 4.03

MGST1 NM_020300 162 100 48 1.76 3.40

NQO1 NM_000903 354 392 105 1.76 3.38

GATA4 AV700724 984 635 327 1.59 3.01

GLRX NM_002064 808 575 328 1.30 2.46

ATP7A NM_000052 1737 1,536 853 1.03 2.04

CHD6 BF572029 457 609 914 -1.00 2.00

HDAC6 NM_006044 895 1,627 1,624 -0.86 1.81

GPX1 NM_000581 5,908 5,570 3,392 0.80 1.74

ARNT NM_001668 380 355 220 0.79 1.73

SIGMAR1 NM_005866 710 550 464 0.62 1.53

CPEB2 AI202327 646 448 425 0.61 1.52

G6PDX NM_000402 612 542 407 0.59 1.50

ATF4 NM_001675 5,980 5,691 3,988 0.58 1.50

BCL2 NM_000633 1,691 1,952 1,130 0.58 1.50

SLC25A24 NM_013386 3,142 3,463 2,151 0.55 1.46

CHCHD4 AI493303 1,303 1,083 907 0.52 1.44

PINK1 AF316873 849 638 592 0.52 1.43

TXN1 AF313911 5,086 4,975 3,840 0.41 1.32

Data shows raw intensity values (Intensity) as well as log2 difference and Fold change between untransfected and High B clone. ND: Not detected

Result in bold refer to gene that was analyzed at protein level by western blot.
